# Genetic portrait of polyamine transporters in barley: insights in the regulation of leaf senescence

**DOI:** 10.3389/fpls.2023.1194737

**Published:** 2023-06-02

**Authors:** Ewelina Stolarska, Umesh Kumar Tanwar, Yufeng Guan, Magda Grabsztunowicz, Magdalena Arasimowicz-Jelonek, Otto Phanstiel, Ewa Sobieszczuk-Nowicka

**Affiliations:** ^1^ Department of Plant Physiology, Faculty of Biology, Adam Mickiewicz University, Poznań, Poland; ^2^ Department of Plant Ecophysiology, Faculty of Biology, Adam Mickiewicz University, Poznań, Poland; ^3^ Department of Medical Education, College of Medicine, University of Central Florida, Orlando, FL, United States

**Keywords:** polyamines, polyamine uptake transporters, bi-directional amino acid transporters, senescence, barley

## Abstract

Nitrogen (N) is one of the most expensive nutrients to supply, therefore, improving the efficiency of N use is essential to reduce the cost of commercial fertilization in plant production. Since cells cannot store reduced N as NH_3_ or NH_4_^+^, polyamines (PAs), the low molecular weight aliphatic nitrogenous bases, are important N storage compounds in plants. Manipulating polyamines may provide a method to increase nitrogen remobilization efficiency. Homeostasis of PAs is maintained by intricate multiple feedback mechanisms at the level of biosynthesis, catabolism, efflux, and uptake. The molecular characterization of the PA uptake transporter (PUT) in most crop plants remains largely unknown, and knowledge of polyamine exporters in plants is lacking. Bi-directional amino acid transporters (BATs) have been recently suggested as possible PAs exporters for Arabidopsis and rice, however, detailed characterization of these genes in crops is missing. This report describes the first systematic study to comprehensively analyze PA transporters in barley (*Hordeum vulgare*, Hv), specifically the *PUT* and *BAT* gene families. Here, seven *PUT*s (*HvPUT1-7*) and six *BAT*s (*HvBAT1-6*) genes were identified as PA transporters in the barley genome and the detailed characterization of these *HvPUT* and *HvBAT* genes and proteins is provided. Homology modeling of all studied PA transporters provided 3D structures prediction of the proteins of interest with high accuracy. Moreover, molecular docking studies provided insights into the PA-binding pockets of HvPUTs and HvBATs facilitating improved understanding of the mechanisms and interactions involved in HvPUT/HvBAT-mediated transport of PAs. We also examined the physiochemical characteristics of PA transporters and discuss the function of PA transporters in barley development, and how they help barley respond to stress, with a particular emphasis on leaf senescence. Insights gained here could lead to improved barley production *via* modulation of polyamine homeostasis.

## 1 Introduction

Nitrogen (N) is one of the most expensive nutrients to supply, and commercial fertilizers represent the major cost in plant production (Bekkerman et al., 2020). Therefore, improving N use efficiency (NUE) is essential to reduce the cost of fertilization. Improved NUE also avoids environmental damage due to nitrate leaching, ecosystem saturation and water pollution. The use of N by plants involves several steps, including uptake, assimilation, translocation, and remobilization (Masclaux-Daubresse et al., 2010). Improving the efficiency of N remobilization (NRE) has the advantage of re-using the N from vegetative parts of the plant for grain filling. In short, NRE improves plant NUE. Until now, most plant cultivars have been selected under non-limiting N conditions for productivity and grain yield. Improved dry matter and yield has been shown to be mainly associated with increased leaf longevity (Duvick, 1992), that is directly linked to leaf senescence (Paluch-Lubawa et al., 2021). To address the question of NRE, efforts have been made to study the biochemical mechanisms involved in N remobilization in order to detect the limiting factors that could be manipulated, as well as to evaluate the genetic basis of NRE and NUE. To facilitate translational research, it is important to develop a good understanding of the complex response(s) that are inherent to leaf senescence- dependent remobilization and catalog them for each crop plant.

In plant biology, polyamines (PAs) and senescence cross paths (reviewed in Sobieszczuk-Nowicka, 2017; Sobieszczuk-Nowicka et al., 2019). PAs are low molecular weight aliphatic nitrogenous bases containing two or more amino groups which are found in different life forms ranging from prokaryotes to eukaryotes (Tabor and Tabor, 1984; Liu et al., 2018; Mustafavi et al., 2018). PAs homeostasis is tightly regulated with intracellular PAs in certain tissues leading to both desired (Wang and Casero, 2006) and undesired phenotypes (Xu, 2015; Sobieszczuk-Nowicka et al., 2019). In plants, putrescine, spermidine and spermine are the major PAs which are involved in the regulation of diverse and important physiological processes (Xu et al., 2014; Xu, 2015; Mustafavi et al., 2018), including senescence, as well as plant responses to biotic and abiotic stresses (Vuosku et al., 2012; Reis et al., 2016; de Oliveira et al., 2017; Mustafavi et al., 2018; Thomas et al., 2020). We found that transformations between individual PAs essentially contribute to dark-induced barley leaf senescence responses (Sobieszczuk-Nowicka et al., 2016). The decrease in free Put throughout senescence is paralleled by the formation of perchloric acid (PCA)- soluble putrescine conjugates that accumulate at high levels in the senescing leaf (Sobieszczuk-Nowicka et al., 2016). Senescence-dependent remobilized nitrogen (N) and carbon (C) flow may contribute to PA conjugation, since the expression of the respective protein-coding genes also increases (Sobieszczuk-Nowicka et al., 2016). As previously discussed, plant cells sense PAs as organic-N and stimulate turnover of N molecules (Mattoo et al., 2010). Correlations between plant production parameters (e.g. grain filling rate and yield components) and cellular PA activity have also been reported (Bányai et al., 2017; Pál et al., 2021).

Polyamine homeostasis is maintained by intricate multiple feedback mechanisms at the levels of biosynthesis, catabolism, uptake, and efflux (Igarashi and Kashiwagi, 2000; Pegg, 2009; Pegg and Casero, 2011). The metabolism of PAs has received great attention in ageing and senescence research, including leaf senescence. In contrast, transport issues are less studied, and these may be the key element in regulation of leaf senescence, and even more broadly, in plant physiology, *via* controlled polyamine redistribution. Indeed, PA transporters provide interesting targets that can be manipulated to assess the genetic basis of nitrogen remobilization efficiency.

Polyamine transporters have been most completely characterized in *Escherichia coli* and *Saccharomyces cerevisiae*. In contrast, not many polyamine transporters have been identified in plant cells so far. In *E. coli* PotABCD and PotFGHI, and in yeast SAM3, DUR3, GAP1 and AGP2 has been characterized as polyamine uptake transporters (PUTs) (Igarashi and Kashiwagi, 2010), and theirs homologs are described as PUTs in plants. The first published record of plant uptake transporter was in 2012 by Mulangi et al. A rice POLYAMINE UPTAKE TRANSPORTER (OsPUT1) was characterized to be a spermidine-specific transporter (Mulangi et al., 2012b). Furthermore, three other rice proteins (OsPUT2, OsPUT3.1, and OsPUT3.2) and three Arabidopsis proteins (AtPUT1, AtPUT2, and AtPUT3) were all confirmed to have polyamine uptake ability (Mulangi et al., 2012a). The rice and Arabidopsis PUTs have been well studied; however, the molecular characterization of the PUT in most other crop plants remain largely unknown.

The knowledge about polyamine exporters in plants is lacking, while PotE and CadB have been reported as PA antiporters in *E. coli* (Schiller et al., 2000; Igarashi and Kashiwagi, 2010). Similarly TPO1-5 were characterized as PA excretion proteins in yeast (Aouida et al., 2005; Uemura et al., 2005; Uemura et al., 2007; Sampathkumar and Drouin, 2015). Based on the homology to these antiporters, bi-directional amino acid transporters (BATs) were identified as PA antiporters in *Arabidopsis* and rice (Ge, 2015; Ariyaratne et al., 2019). In order to demonstrate the exchange of PAs for other amino acids by BATs, individual *BAT* genes (*OsBAT1, AtBAT1.1*, and *AtBAT1.2*) were expressed in the *E. coli* PotE/CadB-double knockout (DKO) mutant (Ge, 2015; Ariyaratne et al., 2019). Export of polyamines from transformed DKO cells was determined by measuring its uptake into inside-out membrane vesicles of the transformed cells and it was suggested that BATs might act as PA exporters (Ge, 2015; Ariyaratne et al., 2019).

With this in mind, we have described the PA transporters (*PUTs* and *BATs*) in barley at the genome scale in our study. First, the genome-wide identification of the PA transporters as well as detailed analyses of their evolution and expression was performed. We also analyzed their physiochemical properties and the role of PA transporters in barley development and stress adaptation with a focus on leaf senescence. In addition, we analyzed the potential regulatory mechanisms controlling the gene expression of PA transporters including promoter analysis and miRNA target sites. Lastly, to confirm the interaction of PAs to the transporters we performed 3D-modeling and molecular docking studies, which suggest that PA transporters have a binding pocket for PAs as ligands.

## 2 Materials and methods

### 2.1 Identification and characterization of polyamine transporter genes and proteins in barley

To identify the putative PA transporter family genes in barley, we used the known sequences of *PUT* and *BAT* genes of Arabidopsis and rice for BLASTp search (E value, 10^−10^) against the barley genome database hosted at Ensembl Plants (https://plants.ensembl.org/index.html). The protein sequences of barley were analysed using Hidden Markov Model (HMM) analysis for the presence of a typical HMM domain representing the corresponding protein class in a HMMER search (https://www.ebi.ac.uk/Tools/hmmer/) (Finn et al., 2011; Upadhyay and Mattoo, 2018). For accuracy, these sequences were also cross verified with InterProScan (Finn et al., 2017), and the candidates containing any of the typical domains were considered as proteins of interest.

Characteristics of identified proteins such as isoelectric point (pI), amino acid sequence length (aa), molecular weight (MW) and grand average of hydropathicity (GRAVY) were obtained using tools available at the ExPASY bioinformatics resource portal (https://www.expasy.org). The sub-cellular localizations were predicted by Plant-mPLoc webtool (http://www.csbio.sjtu.edu.cn/bioinf/plant-multi/). For gene structure analysis, genomic DNA and coding DNA sequences (CDS) corresponding to each identified gene were analysed. The GFF3/GTF annotation file containing the locations of interesting genes in genome and their structural information was extracted from the Ensembl Plants database (https://plants.ensembl.org/index.html). The MEME tool from the MEME suite 5.3.3 (http://meme-suite.org/tools/meme) was used to identify ten statistically significant motifs in the protein sequences based on “zero or one occurrence per sequence (zoops)” (Bailey et al., 2009). Hidden Markov Model analysis was done with HMMER database by the Inter-pro scan program hosted at webtool (http://www.ebi.ac.uk/interpro/). The gene structure, conserved motifs and domains were visualized using the TBtools software (Chen et al., 2018).

### 2.2 Evolutionary analysis of PA transporters in barley

The protein sequences of reported PUTs and BATs in various plant species were retrieved and a phylogenetic tree was constructed using the Neighbor-Joining method with Poisson correction and 1000 bootstrap values using the MEGA-11 program (Tamura et al., 2021). The tree was visualized using the iTOLv6 program (https://itol.embl.de/). The evolutionary distances were computed using the p-distance method and are in the units of the number of amino acid differences per site (Tamura et al., 2021). All ambiguous positions were removed for each sequence pair (pairwise deletion option). Evolutionary analyses were conducted in MEGA-11 (Tamura et al., 2021). To explore the synteny relationships of the orthologous PA transporter genes among barley and other species, genome data and the gene annotation files of *A. thaliana*, *O. sativa*, *B. distachyon*, and *S. bicolor* were downloaded from the Phytozome database (https://phytozome-next.jgi.doe.gov/). The syntenic analysis graphs were constructed by using the Dual Synteny Plotter function in TBtools software.

### 2.3 Promoter analysis, microRNA target site, and protein-protein interaction predictions

To analyse the promoter regions of PA transporter genes, 1.5 kb upstream sequence from the translation start sites was retrieved from Ensembl Plants database. Transcription factor binding sites (TFbs) and *cis*-acting regulatory elements (CREs) were identified within the promoter regions to investigate their role in the regulation of polyamine transporter gene expression. The PlantPAN 3.0 software (http://plantpan.itps.ncku.edu.tw/promoter.php) was used to identify TFbs in the promoter regions of the PA transporter genes. The multiple promoter analysis program (http://plantpan2.itps.ncku.edu.tw/gene_group.php?#multipromoters) was used to identify the common TFbs in different promoter regions. The analysis of CREs was carried out using the PlantCARE database (http://bioinformatics.psb.ugent.be/webtools/plantcare/html/) and visualized with the TBtools software. The prediction of CpG/CpNpG islands and tandem repeats (TRs) in promoter regions was done by PlantPAN 3.0 web server (http://plantpan.itps.ncku.edu.tw/index.html). The coding sequences of barley polyamine transporter genes were analyzed by psRNATarget server (https://www.zhaolab.org/psRNATarget/) for miRNA target sites prediction (Dai et al., 2018). The protein–protein interaction analysis of PA transporters was carried out on the STRING web tool (https://string-db.org/) (Szklarczyk et al., 2021), and the network was generated using Cytoscape-3.9.0 software.

### 2.4 Prediction of PA transporter protein structures, post-translational modifications and molecular docking with PA molecules

The prediction of secondary structure of HvBAT and HvPUT proteins was done using the SOPMA web server (Park and Igarashi, 2013). The three-dimensional (3D) structures of HvBAT/HvPUT proteins were predicted by using Phyre2 server (Seiler and Raul, 2005) and further evaluated with Ramachandran plot, ANOLEA (Atomic Non-Local Environment Assessment) and ProSA analyses. Additionally, the PROCHECK test was used to inspect the 3D structure of PA transporter proteins in the SAVES server (https://saves.mbi.ucla.edu/). The CLICK server was utilized to compare the protein models through the RMSD value calculation based on α-carbon superposition (Moinard et al., 2005). The active sites of HvBAT/HvPUT proteins were predicted through CASTp 3.0 server (Nakanishi and Cleveland, 2021). The post-translational modifications of HvPAT proteins were anticipated using MusiteDeep webserver (https://www.musite.net/) with default parameters. The 3D structures of ligands (PAs) viz., putrescine (C_4_H_12_N_2_; PubChem ID: 1045), spermidine (C_7_H_19_N_3_; PubChem ID: 1102) and spermine (C_10_H_26_N_4_; PubChem ID: 1103) were downloded from PubChem database (https://pubchem.ncbi.nlm.nih.gov). The Autodock 4.2 and Autodock Vina softwares were utilized to prepare the receptor proteins and ligands, and docking simulations, respectively (Wang and Casero, 2006). Subsequently, *HvBAT/HvPUT*-PAs interactions were analysed with PyMOL (https://pymol.org) and visualised with LigPlot^+^ v.2.2.4 software (Mehta et al., 2002).

### 2.5 Expression analysis of PA transporter genes in barley

#### 2.5.1 Spatiotemporal and stress –induced expression analysis

The spatiotemporal and stress- induced expression patterns of barley PA transporter genes were analyzed using mRNA-Seq Gene Level *Hordeum vulgare* (ref: Morex V3) and Affymetrix Barley Genome Array on GENEVESTIGATOR v3 tool (Grennan, 2006; Hruz et al., 2008). The expression data were visualized and heat maps were generated using the TBtools software (Chen et al., 2018) on log2FC scale.

#### 2.5.2 Expression analysis during leaf senescence in barley

RNA-Seq data of barley leaf senescence generated in our lab were utilized (BioProject: PRJNA962050). In brief, for dark-induced leaf senescence (DILS), barley (*Hordeum vulgare* cv. Golden Promise) plants were grown (day/night 16/8 h, light intensity 300 μmol m^−2^ s^−1^) for seven days subsequently senescence was induced by transferring them in darkness. The samples were collected after 0, 4, 7, and 10 days. The experiments were carried out on first leaf of barley. The leaf samples for developmental senescence (DLS) were kindly provided by Prof. Per L. Gregersen (Arhus University, Denmark). DLS included the senescent flag leaves (30 days post anthesis) and control (5 days prior anthesis) leaves. The RNA was isolated in-house as described previously (Tanwar et al., 2022) and sequencing was outsourced to BGI Genomics – Global (Hong Kong). At least three biological and three technical replicates were used. For differential gene expression analysis the STAR-FEATURECOUNT-DESEQ2 pipeline was utilized and the plants at day-0 were considered as a control. Differentially expressed genes (DEGs) were determined using log2FC values and adjusted P value ≤ 0.005 were used to identify up- or down-regulated transcripts.

## 3 Results

### 3.1 Identification and characterization of polyamine transporter genes and proteins in barley

Initially, the identification of PA transporters included nine putative *HvPUT*s and seven *HvBAT*s in barley genome; however two *HvPUT* and one *HvBAT* were excluded from further analyses due to their small/partial available sequences. Three out of seven *HvPUT*s (*HvPUT2, HvPUT3* and *HvPUT6*) are located on chromosome 5, while chromosomes 3, 4 and 6 had one *HvPUT* gene each (**Table 1**). All the *HvBAT*s were located on chromosome 3 except *HvBAT6*, which was present on chromosome 2. The PA transporter proteins ranged in length from 328 (HvPUT5) to 527 (HvBAT6) amino acids, and their molecular weight varied from 34.85 kDa (HvPUT5) to 57.82 kDa (HvBAT6) (**Table 1**). The theoretical *pI* ranged from 5.06 (HvPUT1) to 9.22 (HvBAT2), with an average of 8.04. Considering the stability of the proteins, three members (HvPUT1, HvPUT2 and HvPUT3) had the instability index of more than 40 while the rest of them were inferred to be stable with an instability index of less than 40. Moreover, all the PA transporter proteins had GRAVY values greater than 0, suggesting hydrophobic nature. The majority of HvPUT proteins had a high percentage of aliphatic amino acids, with an average aliphatic index of 104.87, ranging from 98.23 (HvPUT5) to 111.43 (HvPUT3). Furthermore, all the transporter proteins were predicted to be localized in the cell membrane and contained 12 transmembrane domains (TMDs), except HvPUT5 with seven TMDs (**Supplementary Figure S1**, **Supplementary Table S1**).

**Table 1:** Details of identified polyamine transporters in barley.

Ensembl ID	Gene Name	Chromosomal location	Length of Genomic sequence (bp)	Length of Coding sequence (bp)	Length of Protein sequence (aa)	MW in kDa	Theoretical pI	Instability index	Aliphatic index	GRAVY	Sub-cellular	TMD
HORVU.MOREX.r3.6HG0606680.2	*HvPUT1*	6H	2591	1708	525	55.878	5.06	41.13	110.02	0.551	Cell membrane	12
HORVU.MOREX.r3.5HG0439620.1	*HvPUT2*	5H	1976	1885	499	53.034	5.77	42.31	109.88	0.612	Cell membrane	12
HORVU.MOREX.r3.5HG0424430.2	*HvPUT3*	5H	3843	2194	495	54.627	5.56	42.80	111.43	0.593	Cell membrane	12
HORVU.MOREX.r3.7HG0735780.1	*HvPUT4*	7H	2654	1969	488	51.492	8.96	30.21	101.39	0.597	Cell membrane	12
HORVU.MOREX.r3.4HG0412980.1	*HvPUT5*	4H	5145	987	328	34.852	8.63	34.70	98.23	0.355	Cell membrane	7
HORVU.MOREX.r3.5HG0498080.1	*HvPUT6*	5H	3231	1497	498	53.145	7.77	34.21	99.98	0.435	Cell membrane	12
HORVU.MOREX.r3.3HG0223440.1	*HvPUT7*	3H	7905	1470	489	52.492	9.06	35.62	102.99	0.455	Cell membrane	12
HORVU.MOREX.r3.3HG0234440.1	*HvBAT1*	3H	3986	1581	526	56.434	9.01	28.04	107.53	0.622	Cell membrane	12
HORVU.MOREX.r3.3HG0270500.1	*HvBAT2*	3H	3483	1569	522	56.160	9.22	29.93	103.30	0.622	Cell membrane	12
HORVU.MOREX.r3.3HG0319360.1	*HvBAT3*	3H	1914	1569	522	55.638	8.98	22.41	101.69	0.599	Cell membrane	12
HORVU.MOREX.r3.3HG0319380.1	*HvBAT4*	3H	10527	1554	517	55.626	9.00	24.77	105.26	0.604	Cell membrane	12
HORVU.MOREX.r3.3HG0319390.1	*HvBAT5*	3H	8495	1578	525	56.446	9.06	25.12	105.10	0.604	Cell membrane	12
HORVU.MOREX.r3.2HG0169290.1	*HvBAT6*	2H	2923	1584	527	57.822	8.47	31.66	106.58	0.624	Cell membrane	12

**Figure 1:**
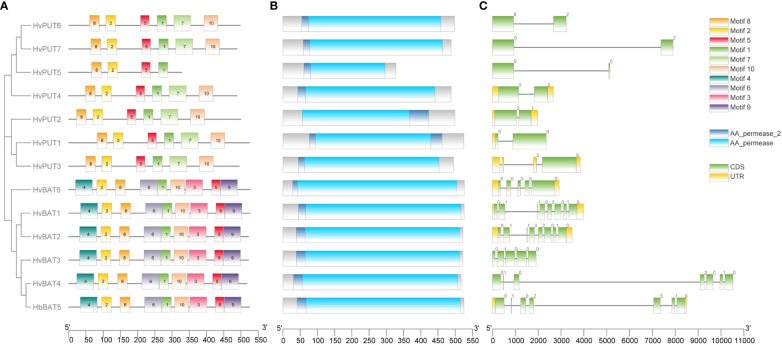
Gene structure and protein conserved motifs of PA transporters in barley. Distribution of 10 conserved MEME motifs as colored boxes in the protein sequences **(A)**. Location of HMM domain in the protein sequences **(B)**. Exon and intron organization of PA transporter genes **(C)**.

Upon further characterization of the PA transporters, we identified the distribution of ten conserved motifs in the studied proteins. The conserved motifs arranged in the order of motifs: 8, 2, 5, 1, 7, and 10 were shared by all HvPUTs except HvPUT5 which did not contain motifs 7 and 10 at the C-terminus (**Figure 1A**). Moreover, all the HvBAT proteins consisted of conserved motifs arranged in the order of 4, 2, 8, 6, 1, 10, 3, 5, and 9 (**Figure 1A**). All the PA transporter proteins consisted of the typical conserved domains for AA_permease_2 (PF13520) and AA_permease (PF00324) (**Figure 1B**). In addition, the PA transporters’ structural diversity was evaluated, and the distribution of exons and introns is shown in **Figure 1C**. Among *HvPATs*, most of the *HvPUTs* had one intron except *HvPUT3* which consisted of three introns in the 5’UTR, while *HvBATs* consisted of 4-7 introns.

### 3.2 Evolutionary analysis of PA transporter genes in barley

**Figure 2:**
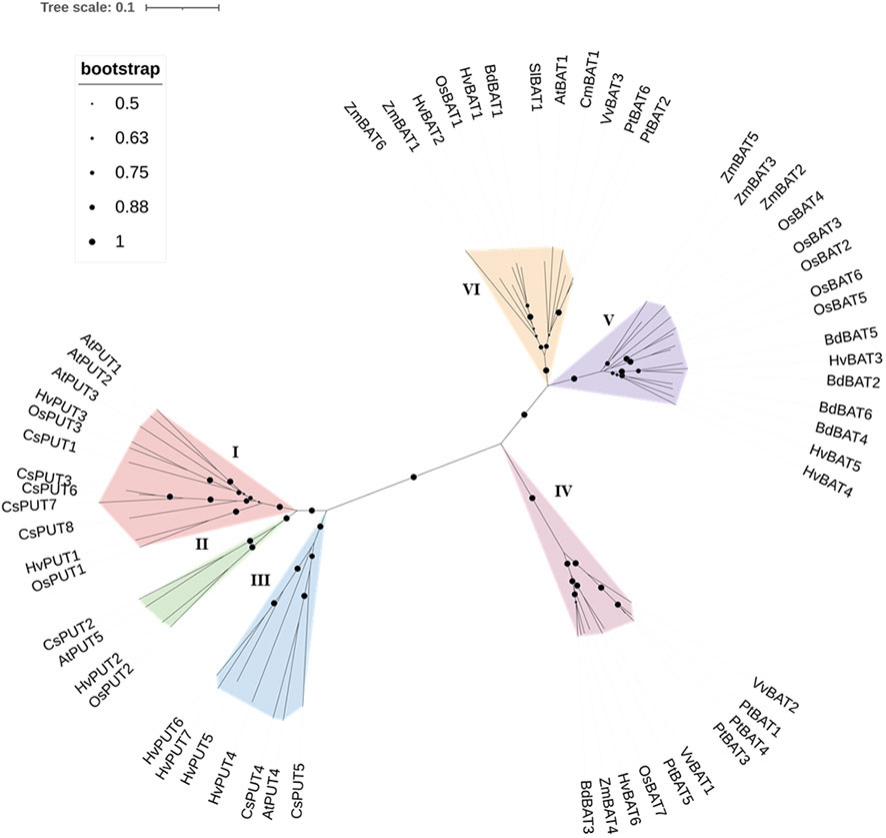
Phylogenetic relationship of PUT and BAT proteins. PUT and BAT protein sequences from *H*. *vulgare* (Hv) and other plant species: *A*. *thaliana* (At), *B*. *distachyon* (Bd), *O. sativa* (Os), *P. trichocarpa* (Pt), *Z. mays* (Zm), *V. vinifera* (Vv), *C*. *sativus* (Cs), *S. lycopersicum* (Sl), and *C*. *melo* (Cm) were used in the analyses. The evolutionary relationships were constructed using the Neighbor- Joining (NJ) method with 1000 bootstrap replications by MEGA-11 software. All 60 proteins are clustered into six groups (three PUTs and three BATs) which are highlighted in different colors.

To establish the evolutionary and phylogenetic relationships of PA transporters, the protein sequences of identified PUTs and BATs from various plant species (**Supplementary Table S2**) were utilized including: Arabidopsis (*Arabidopsis thaliana)*, stiff brome (*Brachypodium distachyon)*, rice (*Oryza sativa)*, poplar (*Populus trichocarpa)*, maize (*Zea mays*), wine (*Vitis vinifera*), saffron crocus (*Crocus sativu*s), tomato (*Solanum lycopersicum*), and melon (*Cucumis melo*). The obtained result indicated that the PUT family divided mainly into three groups (Group I, II, and III) (**Figure 2**). Group I included 12 proteins (including HvPUT1and HvPUT2), four proteins were classified into group II (including HvPUT2), and seven proteins were classified into group III which included HvPUT4-7 (**Figure 2**). Similarly, BATs were also categorized in three groups; HvBAT1 and HvBAT2 were included into group IV, HvBAT3-5 were categorised in group V and group IV contained one HvBAT6. Overall, the HvPUTs and HvBATs showed close association with their homologs in other plant species.

To further understand the evolutionary relationship of PA transporters, collinearity analyses were conducted between barley, Arabidopsis, stiff brome, rice and sorghum (*Sorghum bicolor)* plants. A total of four PA transporters (30.76%) were identified with collinearity relationship in other monocot plant species (**Supplementary Figure S2**; **Supplementary Table S3**). The four orthologous gene pairs (*HvPUT1, HvPUT2, HvBAT3* and *HvBAT6*) were identified from *H. vulgare*-*S. bicolor*, *H. vulgare*-*B. distachyon*, and *H. vulgare*-*O. sativa*. Interestingly, no orthologous gene pair was detected from *H. vulgare*-*A. thaliana.*

### 3.3 Promoter analysis of PA transporter genes in barley

**Figure 3:**
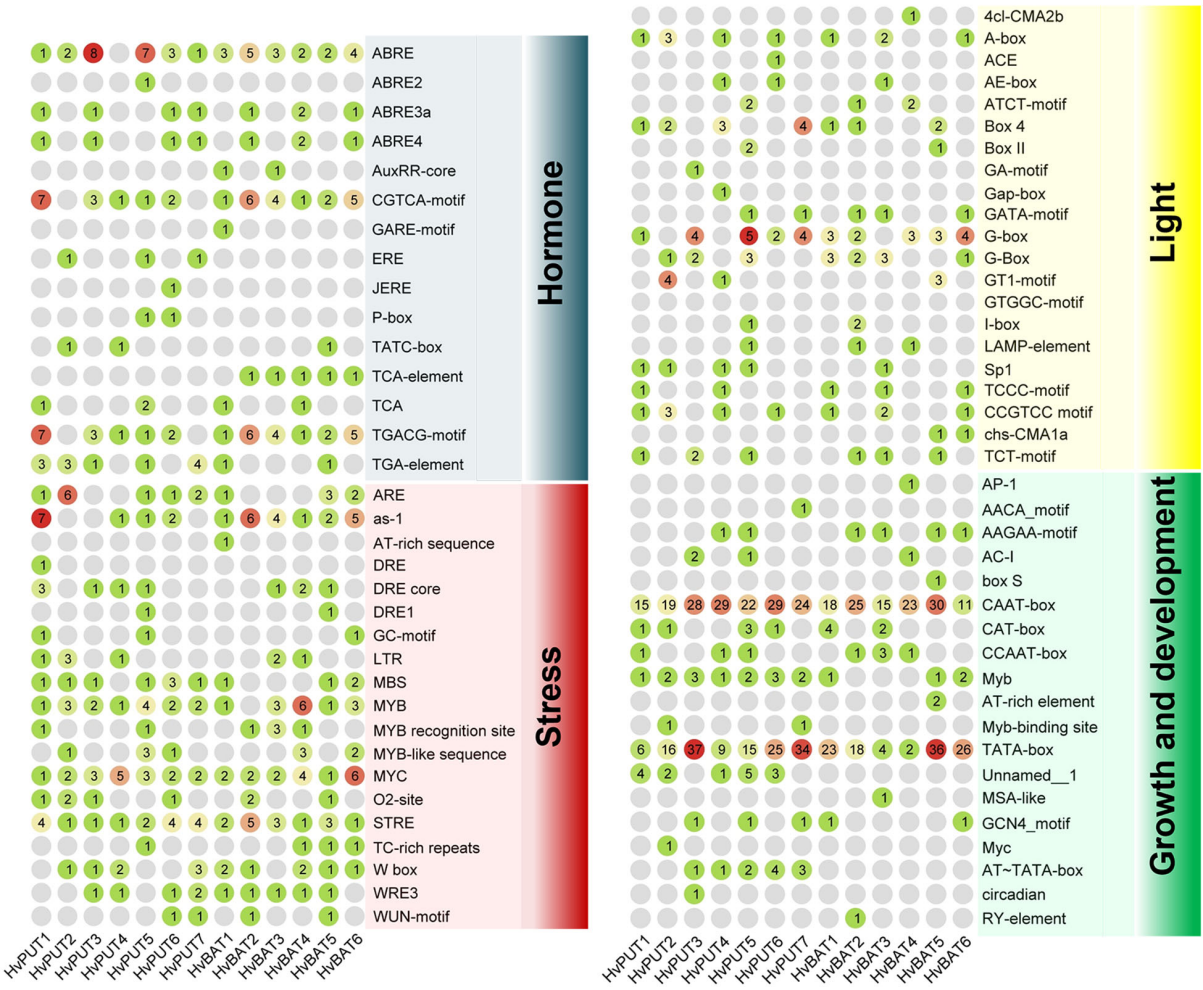
*Cis*-acting regulatory elements identified in the 1.5 kb upstream region of the barley PA transporter genes. The group of elements involved in hormone, stress, light, and growth and development are presented in different colors. The number of elements for each gene are marked in colored circles.

The putative promoter regions (1.5 kb upstream sequences) of the PA transporter genes were analysed for the presence of *cis*-acting regulatory elements (CREs) and transcription factor binding sites (TFbs). A total of 1159 putative CREs were identified in the promoter regions of PA transporter genes. The identified CREs were further divided into four groups:160 elements were related to hormone response, 237 elements associated with stress response, 133 elements related to light response, and 629 elements associated with growth and development (**Figure 3**; **Supplementary Table S4**). CREs for growth and development consisted of 19 elements, among which CAAT-box and TATA-box were the most abundant (288 and 251, respectively), and commonly shared by all of the transporter genes. Moreover, 21 different types of elements were found in the light responsive group, mainly: Box 4, G-box, A-box and CCGTCC elements. Comparatively, 15 types of elements were related to hormone response, mainly ABRE involved in abscisic acid (ABA) response, CGTCA-motif and TGACG-motif in jasmonic acid (MeJA) response, TGA-element and AuxRR-core in auxin response, and ERE in ethylene response. Additionally, stress responsive elements (19 types) comprised STRE (stress-response element), LTR (low temperature response), MBS for drought inducibility, WUN-motif for wound response, and ARE and GC-motif for anaerobic induction.

**Figure 4:**
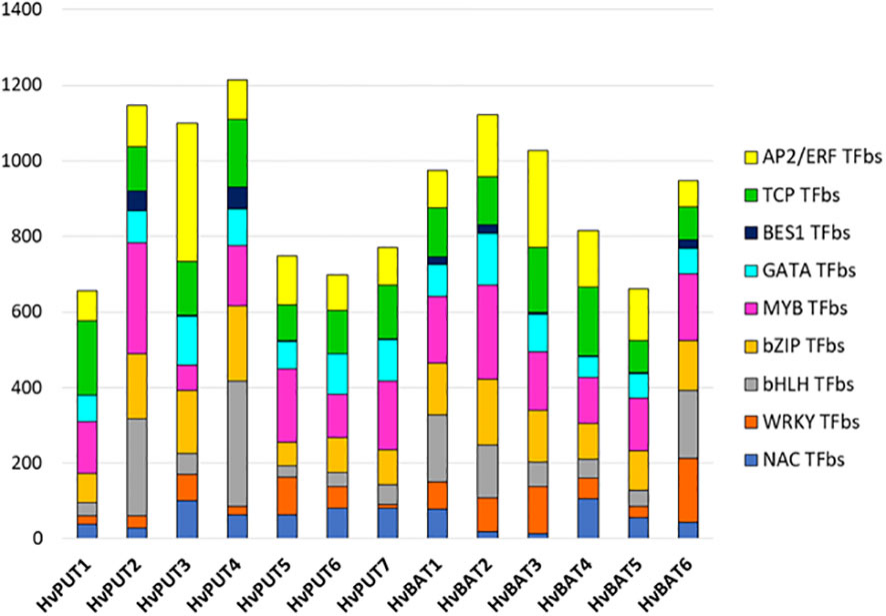
Transcription factor binding site (TFbs) within the promoter regions of PA transporter genes of barley. The different numbers of TFbs in each 1.5 kb upstream promoter sequence of *HvPUT*s and *HvBAT*s genes in barley. Different colors represent different TFbs types.

The putative promoter sequences of seven *HvPUT* and six *HvBAT* genes were used to find common TFbs types, namely TCP, WRKY, bHLH, NAC, BES1, bZIP, MYB, GATA and AP2/ERF. We identified 14,991 and 12,490 TFbs for *HvPUT* and for *HvBAT*, respectively (**Supplementary Table S5**). In *HvPUT* and *HvBAT* gene promoters (**Figure 4**, **Supplementary Table S5**) the TFbs with the highest representation was the MYB-binding site (1151 and 1015, respectively) (**Figure 4**), while the highest number of MYB TFbs was found in *HvPUT2* (292), and *HvBAT2* (249). The second most common TFbs was AP2/ERF-binding site, 988 for *HvPUT* (the highest number was in *HvPUT3* with 366), and 875 for *HvBAT* (*HvBAT3* with 258). The other common TFbs for *HvPUT* was TCP- (981) and bZIP-binding site (861), the highest numbers of these TFbs were present in *HvPUT1* (196), and in *HvPUT4* (201) promoter regions, respectively. While in *HvBAT* promoter regions the other common TFbs was bZIP- (787) and TCP-binding site (784), the highest numbers of these TFbs were present in *HvBAT2* (175), and in *HvBAT4* (183) promoter regions, respectively.

Furthermore, the presence of CpG/CpNpG islands and tandem repeats (TRs) were also analysed in the promoter regions of PA transporter genes. The CpG/CpNpG islands were identified in the 1.5 kb upstream promoter region of four *HvPUT* genes, namely *HvPUT1, HvPUT3, HvPUT4* and *HvPUT7*, and of three *HvBAT* genes: *HvBAT2, HvBAT3* and *HvBAT4* (**Table 2**). TR analysis showed that two *HvPUTs* (*HvPUT6* and *HvPUT7*) contained TRs of 22 and 13 nucleotides in length, respectively, belonging to minisatellites while *HvBAT4* has one TR of two nucleotides long and belong to microsatellites (**Table 3**).

**Table 2:** CpG/CpNpG islands identified in 1.5 kb upstream promoter region of PA transporter genes of barley.

Gene name	Begin site	End site	Length	G+C frequency	CpG o/e ratio	AT Skew	CG Skew
***HvPUT1***	1	664	654	0.52	0.82	0.1	0.02
***HvPUT3***	41	1524	1460	0.54	1.08	0.09	0.02
***HvPUT4***	925	1524	591	0.52	0.94	0.01	-0.08
***HvPUT7***	1	1524	1500	0.54	0.97	0.07	-0.12
***HvBAT2***	746	1373	618	0.49	0.87	0.09	0.19
***HvBAT3***	1	929	914	0.54	0.93	0.19	-0.14
***HvBAT4***	1	940	925	0.5	0.92	-0.22	0.06

**Table 3:** List of promoter regions of the barley PA transporter genes containing tandem repeats.

Gene name	Start	End	Period size	Copy number	% Matches	% Indels	Score	Entropy (0-2)	Seed
***HvPUT6***	882	929	22	2.2	82	10	53	1.79	TGTTGCTATTTGCTCAAACAT
***HvPUT7***	107	134	13	2.2	100	0	56	1.71	TAGTTTATGATAC
***HvBAT4***	1384	1422	2	19.5	100	0	78	1	CT

### 3.4 Prediction of miRNA targets sites and protein-protein interaction analysis

**Figure 5:**
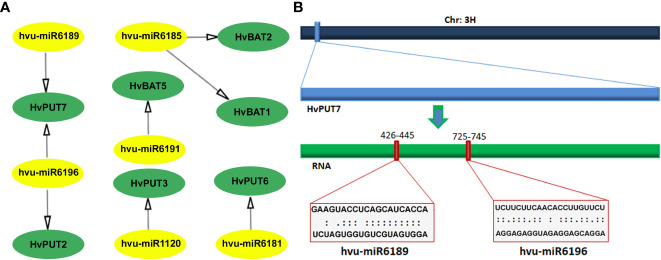
Predicted miRNA target sites in CDS region of PA transporter genes in barley. **(A)** All the predicted miRNAs of *HvPAT* genes; miRNA are shown in the yellow circles, genes are shown in the green circles. **(B)** Scheme of the miRNA targeting site on *HvPUT7.* The navy blue bar represents chromosome 3, the light blue box is *HvPUT7* gene, the green bar represents RNA.

A total of six barley miRNAs (hvu-miR) comprising target sites in the CDS regions of seven PA transporter genes were identified (**Figure 5**; **Supplementary Table S6**). Among *HvPUTs*, The *HvPUT7* consisted of two target sites for hvu-miR6189 and hvu-miR6196, while *HvPUT2, HvPUT3 and HvPUT6* had target sites for hvu-miR6181, hvu-miR1120 and hvu-miR6196, respectively. Moreover, *HvBAT1* and *HvBAT2* had target sites for hvu-miR6185, whilst *HvBAT5* contained target site for hvu-miR6191. Almost all of the identified miRNA-targeted genes, except for *HvPUT7*, were predicted to be silenced by cleavage inhibition. To represent the target accessibility, energy required to unpair the secondary structure around target site (UPE) was also calculated. The results indicated that the UPE varied from 8.657 (hvu-miR6189) to 24.211 (hvu-miR6196) (**Supplementary Table S6**).

**Figure 6:**
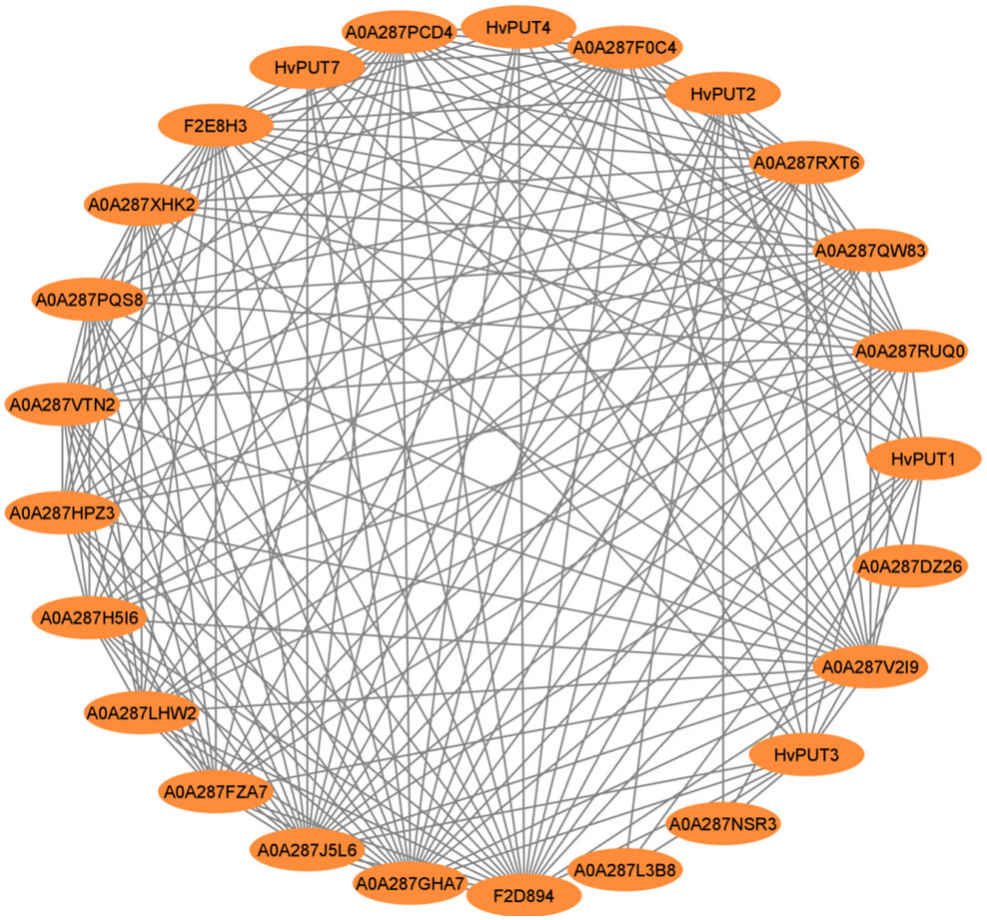
Protein-protein interaction network of PA transporters in barley. Circles represent proteins, lines represent the protein-protein associations.

*In silico* analyses of the protein–protein interactions (PPI) demonstrated that only five HvPUT proteins (HvPUT1, 2, 3, 4 and 7) out of all 13 analyzed (seven HvPUTs and six HvBATs) show protein–protein interactions (**Figure 6**). All the HvPUTs (1, 2, 3, 4 and 7) showed interactions with proteins A0A287F0C4 (Proteasome subunit beta), A0A287GHA7 (26S proteasome non-ATPase regulatory subunit 1 homolog), A0A287J5L6 (PCI domain-containing protein), A0A287RUQ0 (Proteasome subunit beta), A0A287RXT6 (Proteasome subunit beta type), A0A287V2I9 (AAA domain-containing protein), and F2D894 (Proteasome subunit alpha type). Moreover, HvPUT1-3 showed interactions with proteins A0A287DZ26 (DEK_C domain-containing protein), A0A287L3B8 (ABC transporter domain-containing protein) and A0A287NSR3 (DEK_C domain-containing protein). Furthermore, A0A287PCD4 (VWFA domain-containing protein) protein had interactions with HvPUT1, 2, 3, and 7 (**Supplementary Table S7**).

### 3.5 Prediction of proteins structures and post-translational modifications of PA transporters

**Figure 7:**
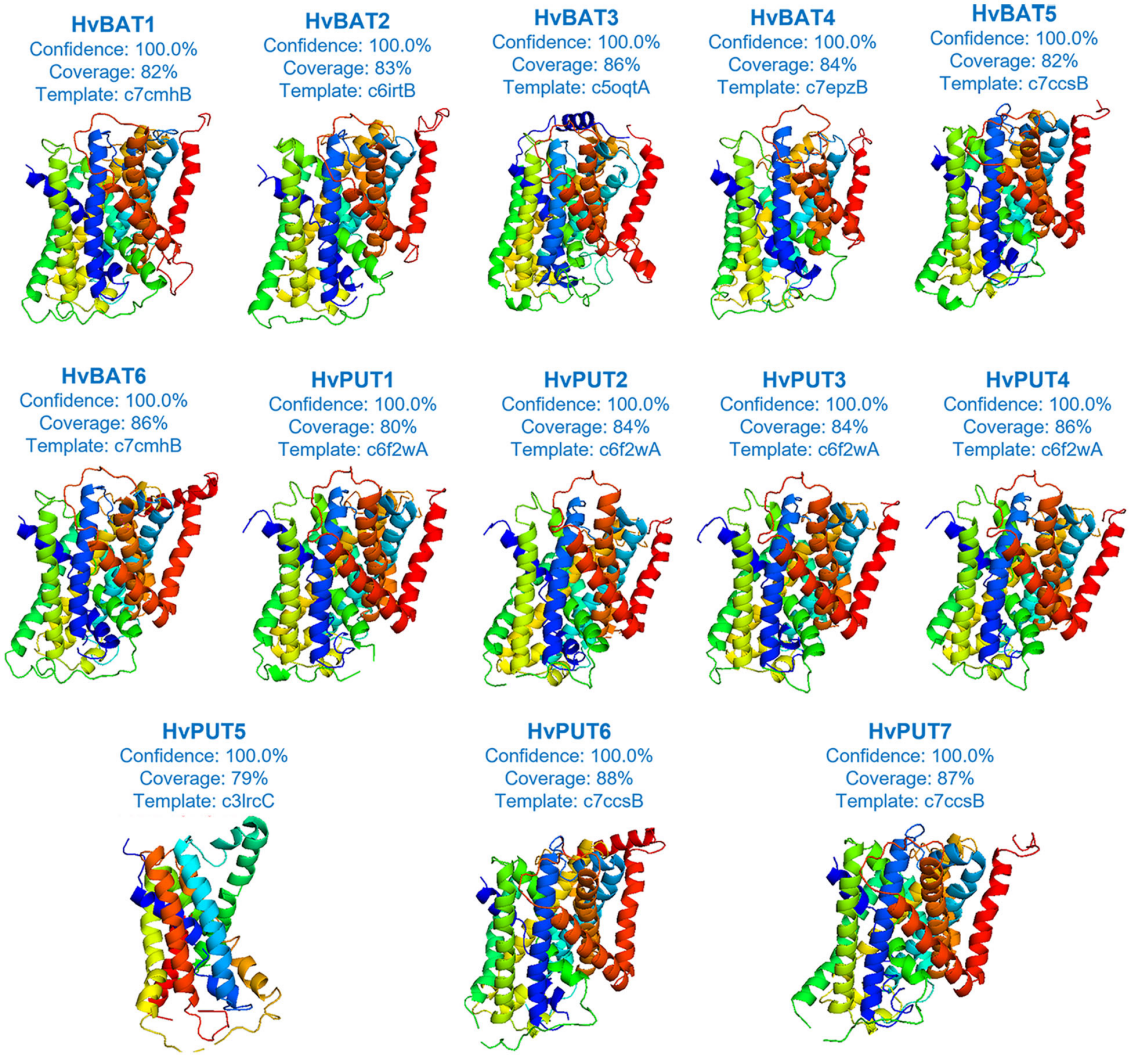
The three-dimensional structures of PA transporter proteins of barley. Each protein structure is accompanied with a percentage of confidence score and coverage with the best available template used for building of 3D models.

Based on our secondary structure analysis of PA transporters, the predominant structure of the HvPUT proteins was alpha helix (42.99-53.91%), followed by random coil (28.46-38.72%), extended strand (13.03-14.75%), and beta turn (3.81-5.94%) (**Supplementary Table S8**). Moreover, the alpha helix in HvBAT proteins ranged from 43.91% to 47.43%, the random coil varied from 30.27% to 33.72%, and the extended strand ranged from 18.03% to 20.50% (**Supplementary Table S8**). Surprisingly, beta turn was only present in HvPUT proteins. The three-dimensional structure of PA transporter proteins was created using the best available template; the confidence in 3D modeling of HvPUT and HvBAT proteins was 100%, while coverage with the best template varied from 79% (HvPUT5) to 88% (HvPUT6) (**Figure 7**; **Supplementary Table S9**).

Ramachandran plot analysis of 3D structures showed that most of the PA transporters had >85%, <16.7% and <1% of residues distribution in most favoured, favoured and disallowed regions, respectively (**Supplementary Figures S3**, **S4**). However, HvPUT5 had 76.2%, 22.4% and 1.4% of residues distribution in most favoured, favoured and disallowed regions, respectively. The ProSA Z-scores of barley PA transporters structures ranged from −3.42 (HvPUT2) to -1.0 (HvBAT2) and the ANOLEA Z-scores ranged from 7.56 (HvBAT6) to 12.22 (HvPUT5) (**Supplementary Table S10**)

We further carried out the analysis for prediction of post-translational modification (PTM) sites in PA transporters of barley. In HvBAT and HvPUT proteins, nine possible PTMs (acetylation, glycosylation, hydroxylation, SUMOylation, methylation, phosphorylation, palmitoylation, ubiquitination, and pyrrolidone carboxylic acid) were predicted (**Supplementary Figure S5**; **Supplementary Table S11**). Most of the PTMs were randomly distributed in the proximity of the N-terminus of all proteins except of HvPUT2 which had PTMs mainly close to C-terminus. The most common PTMs included nine hydroxylation sites (HvPUT1) followed by six phosphorylation sites in HvPUT7 protein, five acetylation sites in HvPUT4 protein, and four glycosylation sites in HvPUTs (1,3,5,6, and 7) and HvBAT (1 and 3) proteins. The SUMOylation site was only predicted in HvPUT3 and HvPUT4, whereas one palmitoylation site was observed in HvBAT (1 and 5) proteins.

### 3.6 Molecular docking of PA transporter proteins

**Figure 8:**
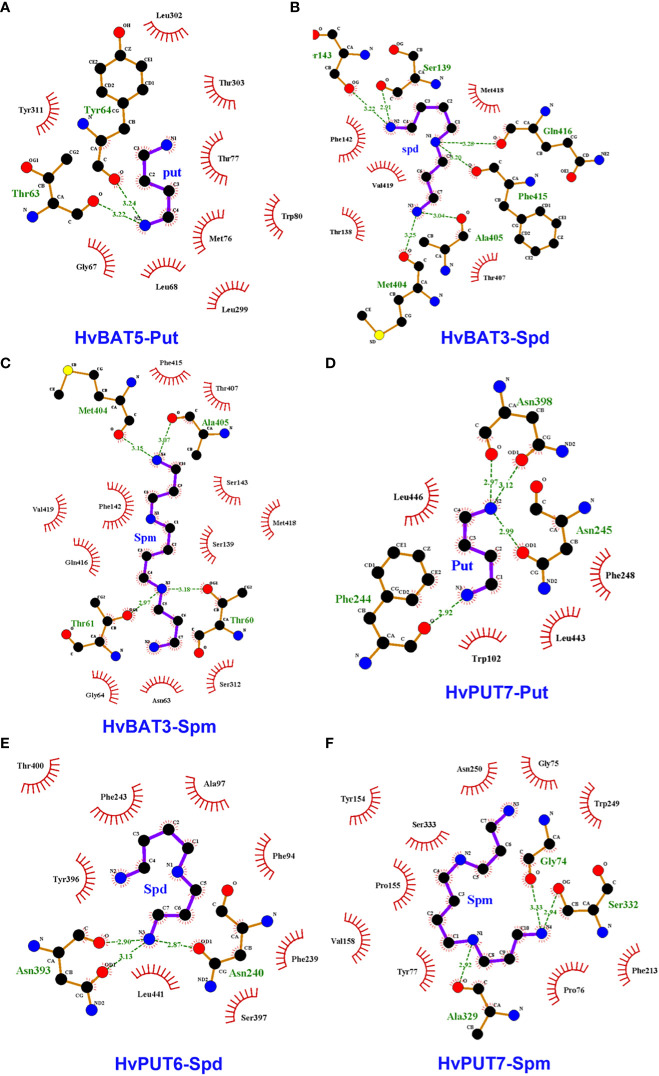
Interactions of polyamines (PAs) in the active sites of the predicted PA transporter proteins’ 3D structures of barley. Diagram shows the binding site of HvBAT and HvPUT proteins with the PA molecules, which are characterized by lowest binding energy and inhibition constant: **(A)** HvBAT5-putrescine, **(B)** HvBAT3-spermidine, **(C)** HvBAT3-spermine, **(D)** HvPUT7-putrescine, **(E)** HvPUT6-spermidine, and **(F)** HvPUT7-spermine. For PAs, carbon atoms are shown in black, oxygens in red, sulfur in yellow, nitrogens in blue. Hydrogen bonds formed between PAs and amino acids are showed in green dashed lines. Amino acids making hydrophobic interactions with the PAs are shown as red arcs with radiating lines. PA atoms involved in these hydrophobic interactions are shown with radiating red lines. Put stands for putrescine, Spm stands for spermine, Spd stands for spermidine. For remaining HvPUT/HvBAT- PAs interactions please see **Supplementary Figures S6**-**S8**.

To explore the interactions of PA molecules in the binding site of predicted PA transporters, we used a molecular docking method. PA transporters showed the lowest mean binding energy (*ΔG*) for spermine (Spm) (*ΔG*: −4.8 kcal/mol; *pki*: 3.56 μmol) compared to spermidine (Spd) (*ΔG*: −4.5 kcal/mol; *pki*: 3.36 μmol) and putrescine (Put) (*ΔG*: −3.8 kcal/mol; *pki*: 2.79 μmol). Among all the HvPUTs, the HvPUT7 showed the lowest *ΔG* with Put (*ΔG*: −4.4 kcal/mol; *pki*: 3.22 μmol) and Spm (*ΔG*: −5.6 kcal/mol; *pki*: 4.11 μmol), while HvPUT6 had the lowest *ΔG* with Spd (*ΔG*: −4.9 kcal/mol; *pki*: 3.59 μmol). Moreover, HvBAT3 showed the lowest *ΔG* with Spd (*ΔG*: −4.9 kcal/mol; *pki*: 3.59 μmol) and Spm (*ΔG*: −5.2 kcal/mol; *pki*: 3.81 μmol) whereas, HvBAT5 showed the lowest *ΔG* with PUT (*ΔG*: −3.8 kcal/mol; *pki*: 2.78 μmol) (**Table 4** **;** **Figure 8**).

**Table 4:** The molecular docking analyses of HvPUT/HvBAT- PAs interactions.

Transporter	Putrescine (Put)			Spermidine (Spd)			Spermine (Spm)		
	ΔG (kcal/mol)	pKi	H-bonds	ΔG (kcal/mol)	pKi	H-bonds	ΔG (kcal/mol)	pKi	H-bonds
HvPUT1	-3.8	2.79	1	-4.7	3.45	2	-4.8	3.52	3
HvPUT2	-4.1	3.01	5	-4.5	3.30	3	-4.7	3.45	4
HvPUT3	-3.7	2.72	4	-4.7	3.45	3	-4.8	3.52	6
HvPUT4	-4	2.94	2	-4.5	3.30	3	-4.7	3.45	3
HvPUT5	-3.2	2.35	4	-3.7	2.72	2	-4.3	3.16	5
HvPUT6	-4	2.94	4	-5	3.67	3	-4.8	3.52	5
HvPUT7	-4.4	3.23	4	-4.9	3.60	5	-5.6	4.11	3
HvBAT1	-3.7	2.72	4	-4.6	3.38	1	-4.8	3.52	1
HvBAT2	-3.7	2.72	3	-4.4	3.23	2	-4.9	3.60	1
HvBAT3	-3.7	2.72	2	-4.9	3.60	6	-5.2	3.81	4
HvBAT4	-3.6	2.64	2	-4.6	3.38	2	-4.7	3.45	2
HvBAT5	-3.8	2.79	2	-4.5	3.30	2	-4.9	3.60	2
HvBAT6	-3.7	2.72	3	-4.6	3.38	4	-4.8	3.52	4

The binding energies (ΔG), inhibition constants (pKi) and the number of hydrogen bonds formed in each protein-ligand interaction (H-bonds) are presented.

In terms of HvPUTs-PA interactions, the number of formed hydrogen bonds varied from one in HvPUT1-Put, HvBAT1-Spd, HvBAT1-Spm and HvBAT2-Spm interactions to six in HvBAT3-Spd and HvPUT3-Spm interactions (**Supplementary Table S12**). HvPUT7 with the lowest *ΔG* showed four hydrogen bonds and four hydrophobic interactions with putrescine, and three hydrogen bonds and ten hydrophobic interactions with spermine (**Figure 8**). HvPUT6 had three hydrogen bonds and eight hydrophobic interactions with spermidine. In case of HvBATs, HvBAT5 with the lowest *ΔG* showed two hydrogen bonds and nine hydrophobic interactions with putrescine. Similarly, HvBAT3 with the lowest *ΔG* showed six hydrogen bonds and five hydrophobic interactions with spermidine, and showed four hydrogen bonds and 11 hydrophobic interactions with spermine (**Figure 8**).

### 3.7 *In silico* expression analysis of PA transporter genes in barley

**Figure 9:**
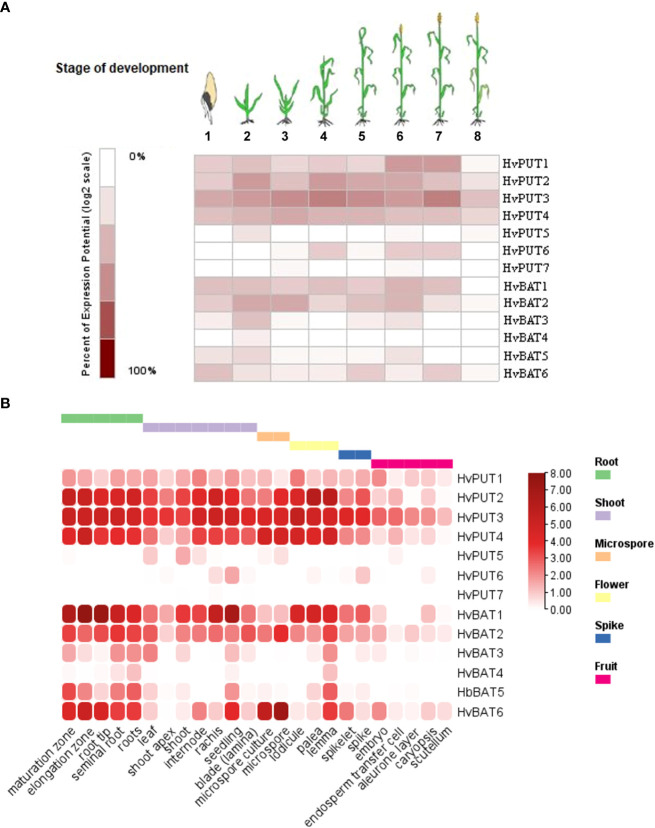
Expression patterns of PA transporter genes in barley plants. **(A)** Diagram showing gene expression pattern of *HvPUTs* and *HvBAT* genes in different developmental stages of barley plants. Eight developmental stages have been analysed as represented: (1) germination, (2) seedling, (3) tillering, (4) stem elongation, (5) booting, (6) flowering, (7) milk, and (8) dough stage. **(B)** Expression pattern of *HvPUTs* and *HvBAT* genes in different plant organs. The colour scale bare represents log2FC values.

To study the possible involvement of specific PA transporters in different phases of development, we have performed gene expression analyses in barley plants. Our studies demonstrate that *HvPUT1-4* show relatively stable and high expression level during plant growth and development stages namely: germination, seedling, tillering, stem elongation, booting, flowering, milk (**Figure 9A**). *HvPUT5* was expressed only at seedling stage, while *HvPUT6* was expressed in stem elongation-, flowering-, and milk stages. Expression of *HvPUT7* was not detected in barley plants at any stage of development. Moreover, *HvBAT1, 2* and *6* were stably expressed during almost whole plant development, while *HvBAT3* and *HvBAT5* were mainly expressed in seedling- and flowering stage, and *HvBAT4* was detected at very low level exclusively in seedlings (**Figure 9A**). Unsurprisingly, all PA transporter genes show very low or undetectable expression in the last stage of barley plant development, namely dough stage.

The organ specific expression analysis (**Figure 9B**) revealed that among *HvPUTs*, only *HvPUT3* is expressed in high level in almost all studied organs, while *HvPUT2* and *HvPUT4* showed high expression in most of the organs, except caryopsis and its elements (i.e embryo, endosperm, aleurone layer and scutellum) as well as in the shoot apex (*HvPUT4*). The other *HvPUTs* showed very low (*HvPUT1, 5* and *6*) or non-detectable (*HvPUT7*) expression in the studied plant organs. Among *HvBATs*, *HvBAT1* and *2* showed high expression in the most of analyzed organs; the exception were caryopsis and its elements, as well as microspore (*HvBAT1*) and leaf (*HvBAT2*). Interestingly, *HvBAT3, 5* and *6* were expressed mainly in roots and seedlings (although the expression of *HvBAT3* was on a very low level), while *HvBAT4* was not detected in any studied organs (**Figure 9B**).

**Figure 10:**
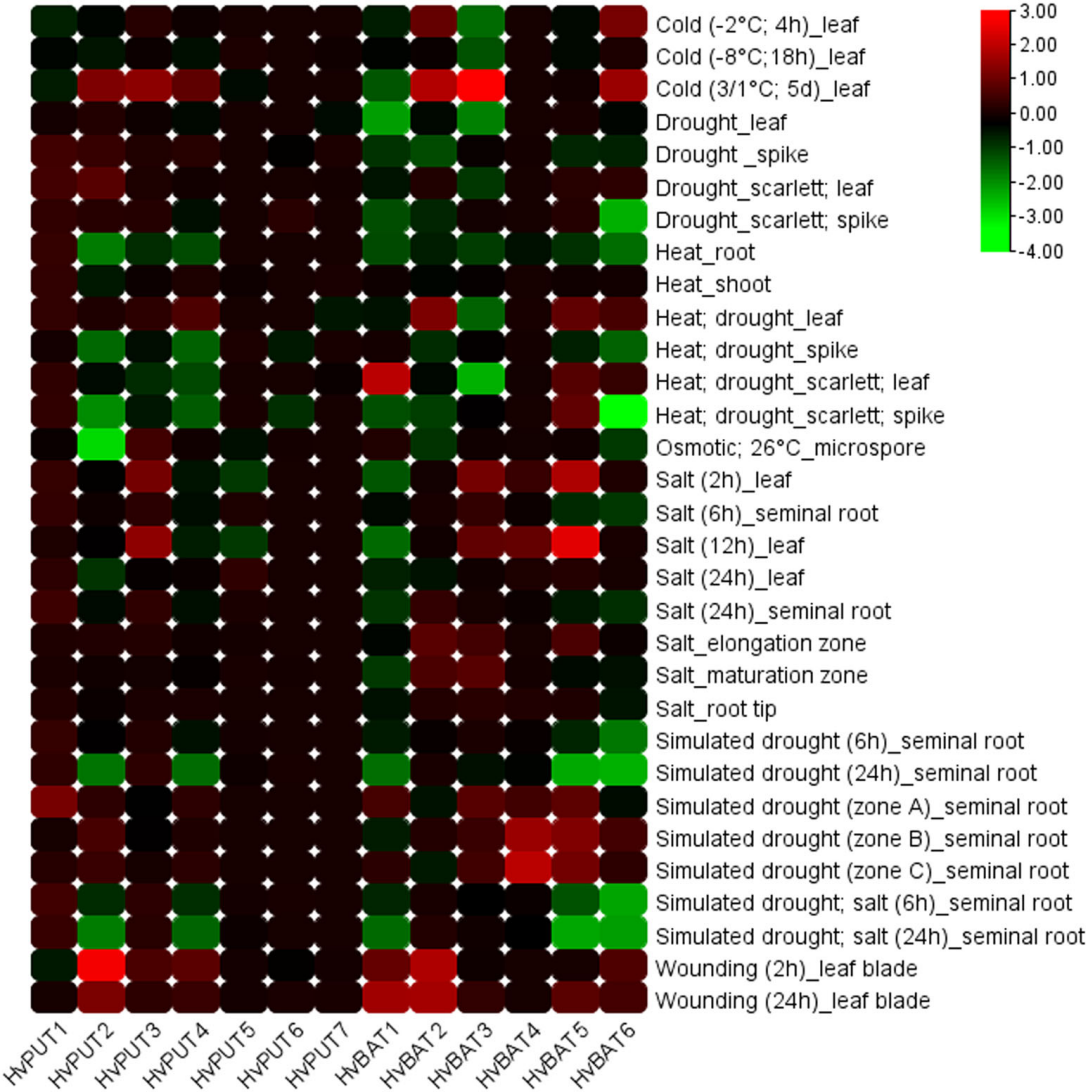
Expression patterns of PA transporter genes in barley plants under various abiotic stress conditions. The color scale represents log2FC values.

Additionally, the expression of PA transporter genes in barley was analysed under various abiotic stress conditions (**Figure 10**). *HvPUT2* was clearly upregulated in wounding stress, and downregulated during cold stress, while the other *HvPUT* genes did not show any significant expression changes in response to abiotic stresses. Moreover, wounding stress resulted in clear upregulation of *HvBAT1* and *2* genes as well as in a slight increase of *HvBAT5* and *6* expression. Simulated drought stress conditions led to decreases in *HvBAT5* and *6* transcript level in barley roots, and was accompanied by upregulation of the *HvBAT4* gene. Moreover, temperature stresses seem to effect *HvBAT*s expression in leaves, leading to upregulation of *HvBAT1,2* and *6* in cold (mainly in prolonged cold-treatment experiments) followed by downregulation of *HvBAT3* and *6* in heat-drought combined stress. Salt treatment showed minor effect on PA transporter genes expression level (**Figure 10**).

### 3.8 Expression analysis of PA transporter genes during leaf senescence in barley

**Figure 11:**
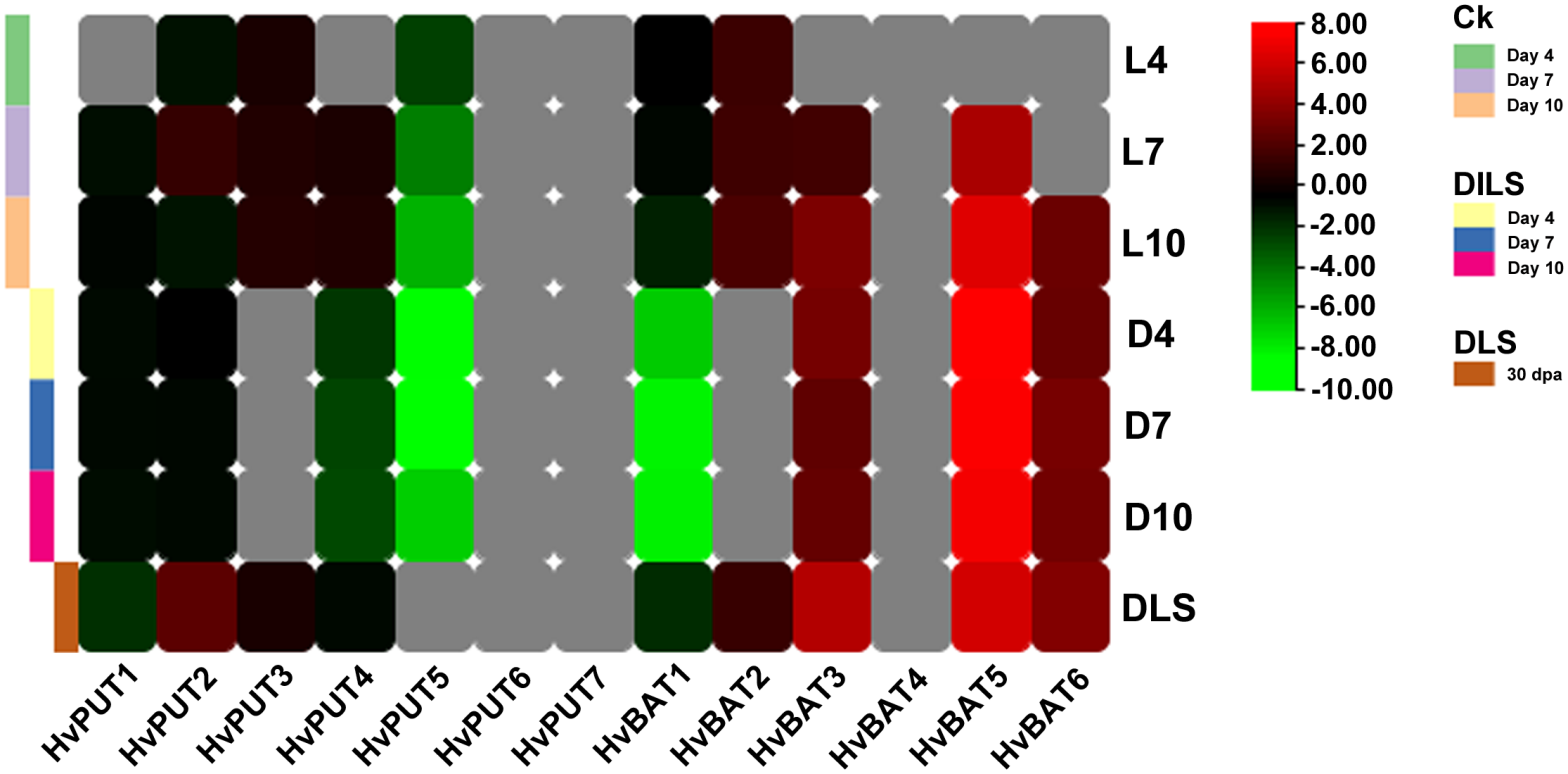
Expression pattern of PA transporter genes during dark- induced leaf senescence (DILS) and developmental leaf senescence (DLS). Heat map showing differences in *HvPUT*s and *HvBATs* expression in barley first leaf on different days of dark induced senescence compared to control plants (plants at day 0) and in a flag leaf during developmental senescence, in its 30^th^- day post-anthesis compared to control (leaf 5 days prior- anthesis). L4- day 4 in light; L7- day 7 in light; L10- day 10 in light; D4- day 4 in dark; D7- day 7 in dark, D10- day 10 in dark; DLS- 30^th^ day post anthesis. Grey color represents expression not detected. Ck represents Control plants.

The RNA- sequencing data developed in our laboratory allowed us to analyze changes in PA transporter gene expression during leaf senescence in barley plants. Clear downregulation of *HvPUT5* expression was observed during the dark-induced leaf senescence (DILS) experiment, while the other *HvPUTs* did not show any significant changes in their expression (**Figure 11**). Moreover, the expression of *HvBAT3, HvBAT6* and *HvBAT5* was upregulated in both DILS- and developmental leaf senescence (DLS) samples. *HvBAT2* had slightly higher expression specifically in DLS and control conditions. The expression of *HvBAT1*, was downregulated during DILS, however, no change was observed in DLS.

## 4 Discussion

### 4.1 Identification and characterization of polyamine transporter genes and proteins

In this work, seven *PUT*s (*HvPUT1-7*) and six *BAT*s (*HvBAT1-6*) genes were identified as putative PA transporters in the barley genome. The average theoretical *pI* value, and the GRAVY values suggest that the majority of PA transporter genes encode alkaline-, hydrophobic- and more likely membranous proteins, that is additionally supported by the subcellular localization predictions as all the PA transporters were localized to the cell membrane. The motif analysis showed that all the PA transporters shared highly conserved corresponding sequences, indicating their similar functionalities. HvPUT5 did not contain motifs 7 and 10 at the C-terminal region. This may be due to partial protein sequence or HvPUT5 may have undergone motif deletion. The protein motif deletion or rearrangement facilitates functional protein diversification, contributing to flexible protein network evolution (Saito et al., 2007). The sequence alignment, protein domains, and motif analysis indicate that the PA transporter genes have shared the same evolutionary pattern and have similar function. All the PA transporters proteins had the signature HMM AA_permease_2 (PF13520) and AA_permease (PF00324) confirming the amino acid transporter activity. The PA transporters’ structural diversity was evaluated by assessing the distribution of exons and introns. The variation in the number of introns is anticipated because the number and length of introns in genes vary depending on organism and gene structure, and these differences may be related to intron function (Grabherr et al., 2011). Moreover, the gain and loss of introns can change the structure of genes and play a vital role in the evolution of gene families (Xu et al., 2012). *HvPUT7* and *HvBAT4* had the largest intron, it can be assumed that these genes served as a precursor from which the other members originated by undergoing an ‘intron loss’ event through the time of evolution (Gilbert et al., 1997). Also, *HvPUT5* has a large intron but a very short second exon, which may confirm the conjecture made earlier that this may be due to partial protein sequence. We note that *HvPUT3* had three introns in the 5’UTR which depicts its uniqueness among all the *HvPUT* members. We hypothesize that this gene structure might be involved in the regulation of gene expression or RNA stability (Shi et al., 2020).

### 4.2 Evolutionary analysis of PA transporters in barley

Our results divided the PA transporters in different groups and the barley PA transporters were clustered together with other plants transporters implying that they may serve similar functions to their plant homologs. The barley genome had a different number of homologs for PA transporters as compared to Arabidopsis implying that the barley PA transporter gene subfamilies may have undergone gene expansion and/or gene loss during the course of its evolutionary history.

To further investigate the evolutionary relationships of PA transporter genes, we performed genome-to-genome synteny analysis between barley and four representative plant species. Comparatively, the PA transporter genes in Arabidopsis did not show any orthologous correlation with barley. However, the monocotyledons showed a considerable synteny. Therefore, we speculated that the syntenic correlations between PA transporters might be connected to the species’ evolutionary divergence. In particular, four PA transporters were identified to be syntenic across all the monocotyledons tested, indicating that these orthologous pairs are conserved and may have existed before the species divergence. We note that the intersections of syntenic PA transporters among different species may be useful for undertaking future relevant gene evolution studies.

### 4.3 Promoter analysis of PA transporter genes in barley

We investigated the potential regulatory mechanism that controls the expression of PA transporter genes in barley by looking into *cis*-acting regulatory elements (CREs), transcription factor binding sites (TFBs) and CpG/CpNpG islands in the promoter regions. Here, we identified a total of 1159 putative CREs taking part in multiple biological processes and found a high number of CAAT-box and TATA-box elements in the promoter regions of PA transporters. Additionally, a large quantity of light-, hormone-, and abiotic stress-responsive elements in the promoter regions were identified which imply that the expression PA transporters might be controlled by a number of different elements which act through several pathways. We further identified the TFbs and classified as common TFbs (TCP, WRKY, bHLH, NAC, BES1, bZIP, MYB, GATA and AP2/ERF). These results further support the CREs analysis that expression of PA transporters might be controlled by a number of different elements. For instance in our study, the MYB-binding site was the most abundant TFbs in all barley PA transporter gene promoters. The highest number of MYB TFbs was found in *HvPUT2* gene promoter, therefore, it may be strong candidate for MYB TFbs-mediated barley development control.

The presence of CpG/CpNpG islands in promoter regions represents critical sites for DNA methylation, which resulted in gene silencing (Cao and Jacobsen, 2002; Zemach and Grafi, 2007). The CpG/CpNpG islands were identified in the promoter region of four *HvPUTs* and three *HvBATs*, implying that expression of these genes might be controlled by DNA methylation. Overall, the study of CREs, TFbs and CpG/CpNpG islands suggested that promoters of PA transporter genes may play important roles in modulating the gene expression during developmental processes and/or stress tolerance. Further, we analysed the tandem repeats (TRs) in the promoter regions. In the promoter sequences of the genes we studied, TRs were found only in *HvPUT6*, *HvPUT7* and *HvBAT4*. This indicates that in these genes there is a higher probability of mutation accumulating during replication (known as polymerase slippage) (Ahmar and Gruszka, 2022). The presence of TRs in these promoters may be useful for mutational analysis, and it may have a role in controlling gene expression of PA transporters.

### 4.4 MicroRNA and protein-protein interaction analysis of PA transporter genes

Many reports have shown that miRNAs regulate responses of barley to different stress conditions (Kantar et al., 2010; Hackenberg et al., 2012; Sunkar et al., 2012; Wu et al., 2018). In this study, six barley miRNAs with target sites in CDS of seven PA transporters were identified. These miRNAs might be involved in various stress responses in barley. For instance, upregulation of hvu-miR6196 occurs during salt adaptation of the autopolyploid *Hordeum bulbosum* (Liu and Sun, 2017) and during barley exposure to an excess of boron (Unver and Tombuloglu, 2020). In this study, hvu-miR6196 had target sites in two transporters; *HvPUT2* and *HvPUT7* implying that their expression might be regulated by miRNA during the various stress conditions. Thus, exploring the role of miRNAs in PA transporter gene functions in response to various stresses would be of great interest.

Protein–protein interactions (PPI) represent an important aspect of plant systems biology which enables uncovering the unknown functions of proteins at the molecular level and can provide insight into complex cellular networks. In this study, the regulatory PPI network for the barley PA transporter proteins indicated considerable interactive networks among HvPUTs and several other proteins. This result implies that PUTs take part in protein complexes which have essential roles in regulatory processes, cellular functions and signaling cascades. For instance, all the HvPUTs showed PPI with proteasome subunits (alpha/beta). Interestingly, HvBAT protein did not show any PPI, suggesting that HvBATs are not involved in the regulatory protein complexes which have essential roles in physiological processes. Alternatively, the possibility exists that the database for PPI network of BATs has not been fully established making this type of interpretation more challenging.

### 4.5 Homology modeling and molecular docking of PA transporter proteins

In this study, we carried out homology modeling of the PA transporter proteins, and the 3D structures of all the proteins showed the high accuracy of the structures predicted. ProSA Z-scores analysis revealed the structures were typically plotted within the range of scores for native proteins of similar size from X-ray crystallography and NMR sources, suggesting no significant deviation from the native structures. Furthermore, we predicted the post-translational modifications (PTMs) in the protein sequences of barley PA transporters. Protein PTMs significantly increase proteome diversity, increase functionality, and allow for rapid responses, all at a low cost to the cell. The commonly found PTM sites were glycosylation and phosphorylation and were present in all the PA transporters. Our results suggested that barley PA transporters might play various roles in development and growth, and various stress conditions through PTMs. Interestingly, some PTM sites were found on specific transporters such as SUMOylation sites were only predicted in HvPUTs (3 and 4), palmitoylation sites were observed in HvBATs (1 and 5), suggesting their specific roles in plant physiology.

Probing the PAs binding pockets of barley PA transporter proteins through molecular docking facilitated an *in silico* understanding of the mechanisms and interactions involved in HvPUT/HvBAT-mediated PAs transport. Weak intermolecular interactions such as hydrogen bonding and hydrophobic interactions are key players in stabilizing energetically-favored ligands, in an open-conformational environment of protein structures (Patil et al., 2010). The numbers of hydrogen bonds in the PA transporter-PA complex were less than the number of hydrophobic interactions in all protein-ligand complex. Our results showed that the most suitable ligand-protein interaction included HvPUT7-Put/Spm, HvBAT3-Spm/Spd, HvBAT5-Put, and HvPUT6-Spd. However, further experimental analysis should be done to verify these predictions. Overall, these results provided that the interacting residues are functionally conserved and have important considerations for protein activity while designing PA transporters modified by genetic manipulation in barley.

### 4.6 Expression analysis of PA transporter genes in barley

The organ-specific expression analysis showed that mRNA of three out of the seven identified barley PUT genes, namely *HvPUT2*, *HvPUT3*, and *HvPUT4*, were clearly detectible in a majority of plant organs. The observation that three listed above may be the main transporters responsible for PA uptake in plants is additionally supported by the relatively stable expression pattern of these three genes during plant growth and development. Interestingly, *HvPUT1* and *HvPUT6* genes characterized by low expression in different barley organs, show transient upregulation during flowering and early seed development suggesting their specific functions in these processes. Among all PA transporters, *HvPUT7* was rarely expressed in all the analysed organs and developmental stages. The archived down-regulation in some gene expressions is fundamental for keeping up with the gene duplicates and ancestral functions (Qian et al., 2010). Thus, the archived down-regulation of *HvPUT7* expression in barley might be regarded as critical for maintaining their biological processes and protecting them from any misfortune during cell crisis. Our study showed that *HvBAT3* and *HvBAT5* were mainly expressed in seedling and flowering stage, suggesting their role in these stages. *HvBAT1*, *HvBAT2* and *HvPUT4* showed high expression in most of the organs except endosperm, shoot apex, embryo, caryopsis, aleurone layer and scutellum.

Changes in the PA pools in plants under stress conditions are well documented and have been reviewed (Wang et al., 2003; Wang and Casero, 2006; Nakanishi and Cleveland, 2021; Pál et al., 2021). Several PA biosynthesis genes were highly upregulated by cold, salt and/or drought stress and, as a result, cellular PA concentrations were increased in Arabidopsis under these stress conditions (Urano et al., 2003; Hummel et al., 2004; Urano et al., 2004; Vergnolle et al., 2005; Alcázar et al., 2010). The question remains whether the expression of PA transporters may be also altered in response to stresses in order to fine tune the optimal PA concentration in the cell or to improve intra/extra cellular transport rates to maintain homeostasis. In our analysis, *HvPUT2* was upregulated in wounding stress, while downregulated during cold stress, suggesting its role in these stresses. *HvBAT3* was upregulated in cold stress and *HvBAT4* had high expression under simulated drought stress. HvBAT5 showed higher expression in salt stress, and *HvBAT6* was downregulated in drought, heat;drought and simulated drought stresses.

Furthermore, in this study we analysed the expression of PA transporters during leaf senescence (DILS and DLS) in barley. Our results showed that *HvPUT4* was slightly and *HvPUT5* was strongly downregulated in DILS. The expression of *HvPUT1* and *HvPUT2* has not changed during DILS. The expression of *HvBAT3*, *HvBAT6* and *HvBAT5* was upregulated in both, DILS and DLS. PUTs are mainly responsible for PA uptake and BATs may act as exporters of PAs, as mentioned before. Thus, we speculated that in senescing cells the uptake of PAs was halted and they mainly relied on the biosynthesis of PAs, which is also supported by findings that PA biosynthetic genes are upregulated during leaf senescence in barley (Sobieszczuk-Nowicka et al., 2016; Tanwar et al., 2022). PA transporters might play important roles in the regulation the leaf senescence by regulating cellular PA homeostasis. The upregulation of *HvBAT*s suggest that PAs may have possible roles in N remobilization to other cells/organs.

The molecular processes underlying leaf senescence are strongly conserved between plant species, suggesting that senescence has evolved as a selectable trait in plants. The phenomenon of senescence is often portrayed as a paradox, as this trait promotes the death of the individuals. This view is too simplistic, as plants are not slated to die before they undergo successful reproduction. Senescence is essential for sustaining the phenotypic plasticity of growth, and it represents an important evolutionary trait that enables plants to adapt to the environment. The impact of senescence on crop yield and quality, and its potential use in breeding more environmentally-resilient plants, are becoming increasingly important. An important aspect of that is the adequate uptake and remobilization of N that increases the plant’s N usage efficiency, thereby reducing the requirement for fertilizers (Schippers et al., 2015; Paluch-Lubawa et al., 2021). Since cells cannot store reduced N as NH_3_ or NH_4_^+^, PAs are important N storage compounds in plants (Wuddineh et al., 2018).

## 5 Conclusions

In this work polyamine transporter genes were identified and characterized at a whole-genome scale in barley to provide insights into their genetics, regulatory framework, physicochemical properties, phylogenetic and evolutionary relationships, and expression profiles during developmental stages in different tissues, and under abiotic stresses with special emphasis on leaf senescence. This is the first systematic study to comprehensively analyze the barley PA uptake transporter gene families and the first systematic study to comprehensively analyze the PA export transporter gene families in plants.

Plant PA transporters and their effector molecule(s) should be an area of future scientific focus because the crosstalk among these molecules likely affects senescence-dependent N remobilization. Understanding the importance of compartmentation is also essential to study the function and mechanism of enzymes and transporters at subcellular level. In the complex organization within the cell, active molecules are usually synthesized in one compartment and then relocated to another for their designated functions (Sweetlove and Fernie, 2013). For example, in human pancreatic cancer cells the polyamine transport protein ATP13A3 changes its localization pattern under conditions of polyamine depletion from the nucleolus to the plasma membrane to facilitate polyamine uptake (Madan et al., 2016; Sekhar et al., 2022).

Future studies will, therefore, need to look at both relative protein expression patterns and protein localization as a function of cellular stress to see if plants and humans use similar strategies to regulate polyamine uptake.

## Data availability statement

Publicly available datasets were analyzed in this study. This data can be found here: https://plants.ensembl.org/index.html.

## Author contributions

ES-N and UT conceived of the presented idea. ES, UT, and YG performed the bioinformatics analysis, and analyzed the data. ES-N and UT designed and supervised the study and interpreted the results. ES, UT, MG, and ES-N drafted the manuscript. MA-J and OP gave suggestions on bioinformatic and experimental analysis and contributed to writing the manuscript. All authors contributed to the article and approved the submitted version.

## Supplementary material

Supplementary Table 1 Details of predicted transmembrane domains in HvBAT and HvPUT proteins sequences in barley.

Supplementary Table 2 Accession numbers of BAT and PUT family from various plants used in this study.

Supplementary Table 3 Synteny analysis of PA transporter genes in *H. vulga*re with other plant species, *A. thaliana*, *B. distachyon*, *O. sativa*, and *S. bicolor*.

Supplementary Table 4
*Cis*-acting regulatory elements identified in the 1.5 kb upstream region of the barley PA transporter genes.

Supplementary Table 5 Transcription factor binding site within the promoter regions of PAs transporter genes of barley.

Supplementary Table 6 Details of predicted miRNA target sites in CDS region of PA transporter genes in barley.

Supplementary Table 7 Protein-protein interaction network of PA transporter genes in barley.

Supplementary Table 8 Secondary structure prediction of PA transporters in barley.

Supplementary Table 9 3D modelling results of PA transporter proteins of barley.

Supplementary Table 10 Quality of the 3d-structures of PA transporter proteins of barley.

Supplementary Table 11 Prediction results of post-translational modification sites in PA transporter protein sequences of barley.

Supplementary Table 12 The snapshot of PA transporters docking with PA molecules. The molecular docking showing the amino acid residues forming hydrogen and hydrophobic interactions with four Pas molecules viz., putrescine, spermidine and spermine.
