# Supplementary material for: Genetic portrait of polyamine transporters in barley: insights in the regulation of leaf senescence
Source: Front Plant Sci. 2023 Jun 2;14:1194737. doi: 10.3389/fpls.2023.1194737 (PMC10272464; doi:10.3389/fpls.2023.1194737)
Supplement: Supplementary file 1 [file DataSheet_1.docx]

*Supplementary Material*

**Genetic portrait of polyamine transporters in barley: insights in the regulation of leaf senescence**

Ewelina Stolarska, Umesh Kumar Tanwar, Yufeng Guan, Magda Grabsztunowicz, Magdalena Arasimowicz-Jelonek, Otto Phanstiel IV, Ewa Sobieszczuk-Nowicka*

* Corresponding author

E-mail address: ewa.sobieszczuk-nowicka@amu.edu.pl

1. **Supplementary Figures**


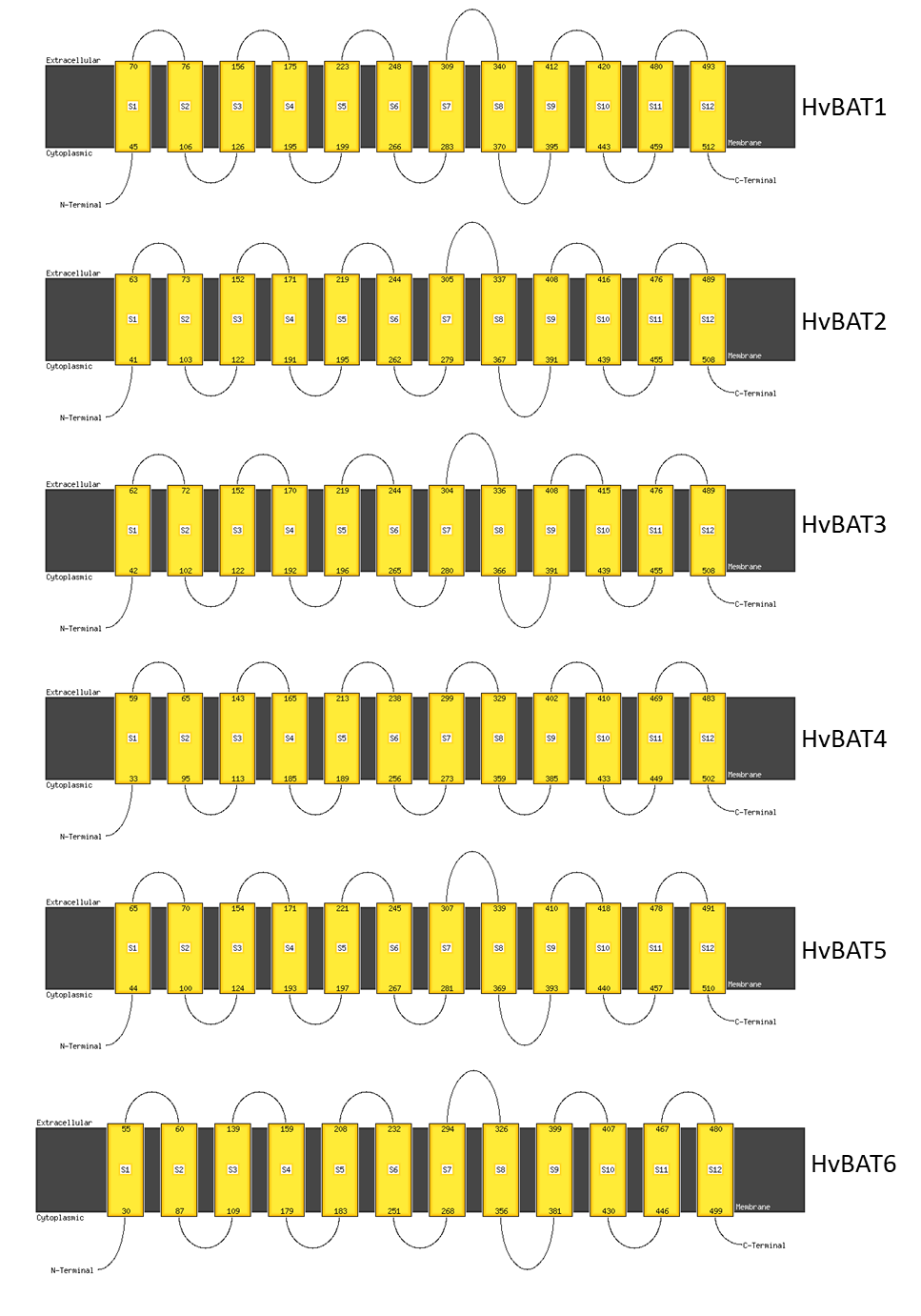

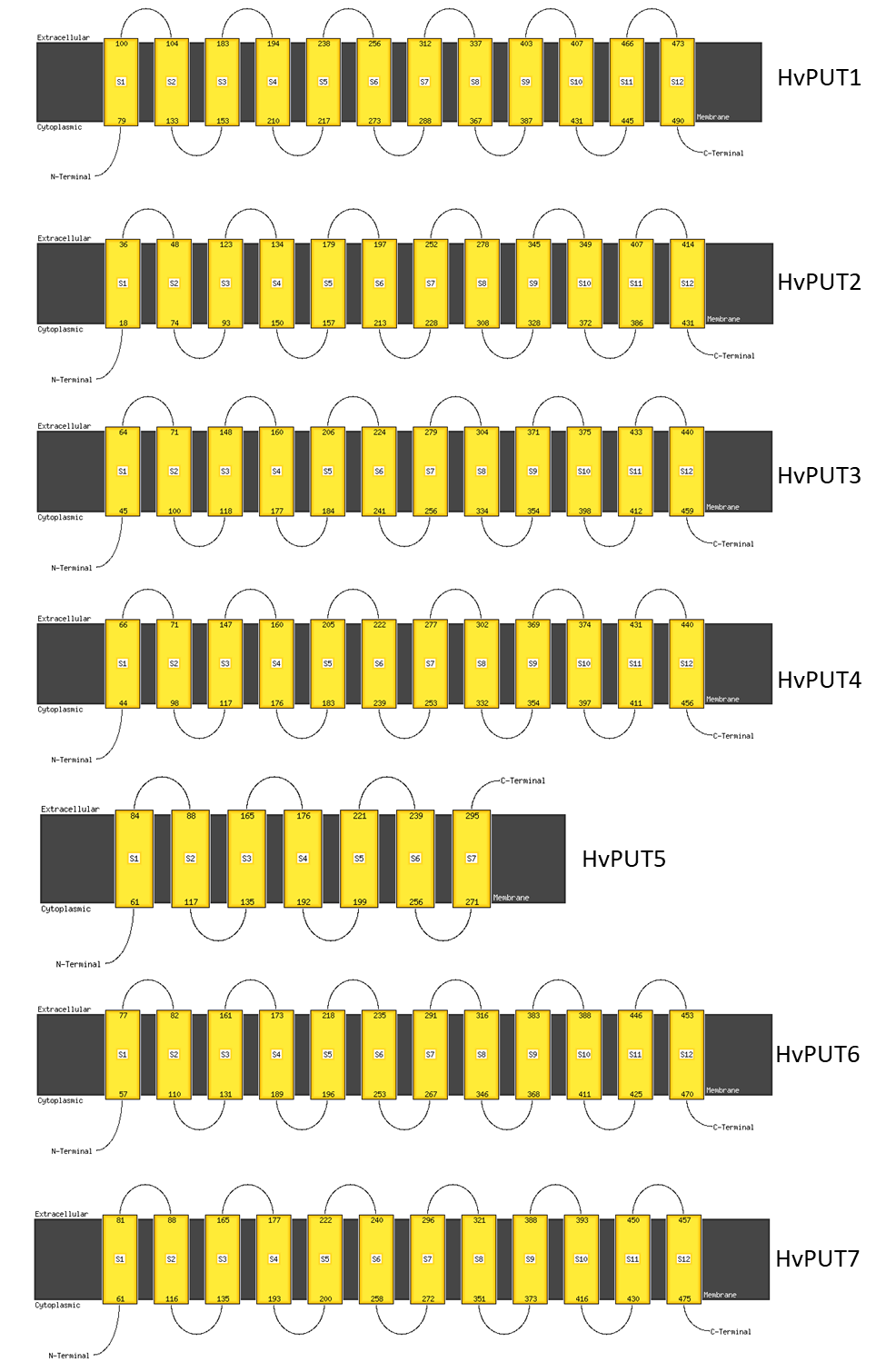


**Supplementary Figure S1.** Instance of prediction of transmembrane domains in HvBAT and HvPUT proteins


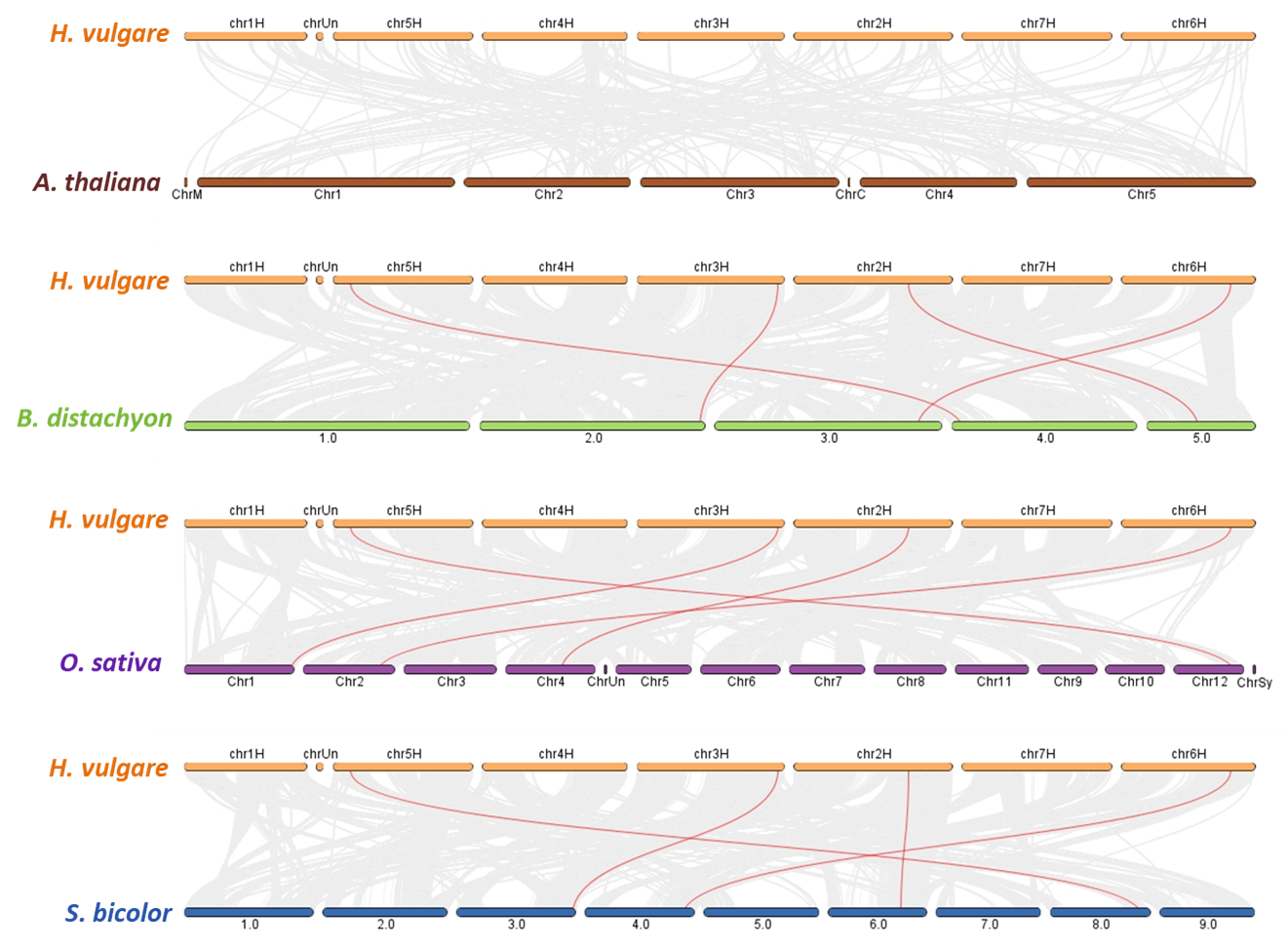


**Supplementary Figure S2.** Synteny analysis of PA transporter genes in *H. vulgare* with other plant species, *A. thaliana, B. distachyon, O. sativa*, and *S. bicolor*. Gray lines in the background indicate the collinear blocks within *H. vulgare* and other plant genomes, while the red lines highlight the syntenic PA transporter gene pairs.


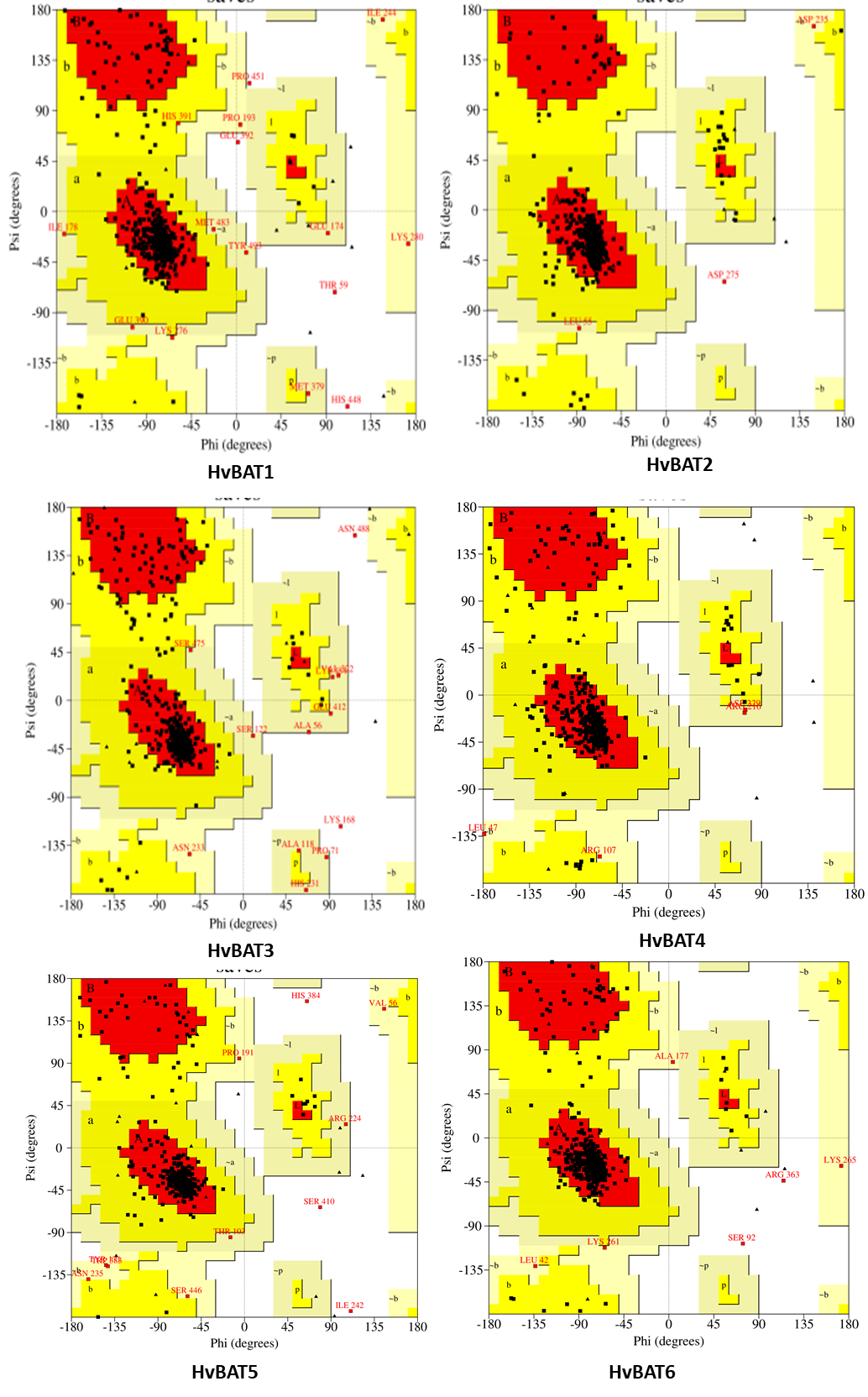


**Supplementary Figure S3.** Ramachandran plot analysis of homology models of HvBAT proteins.


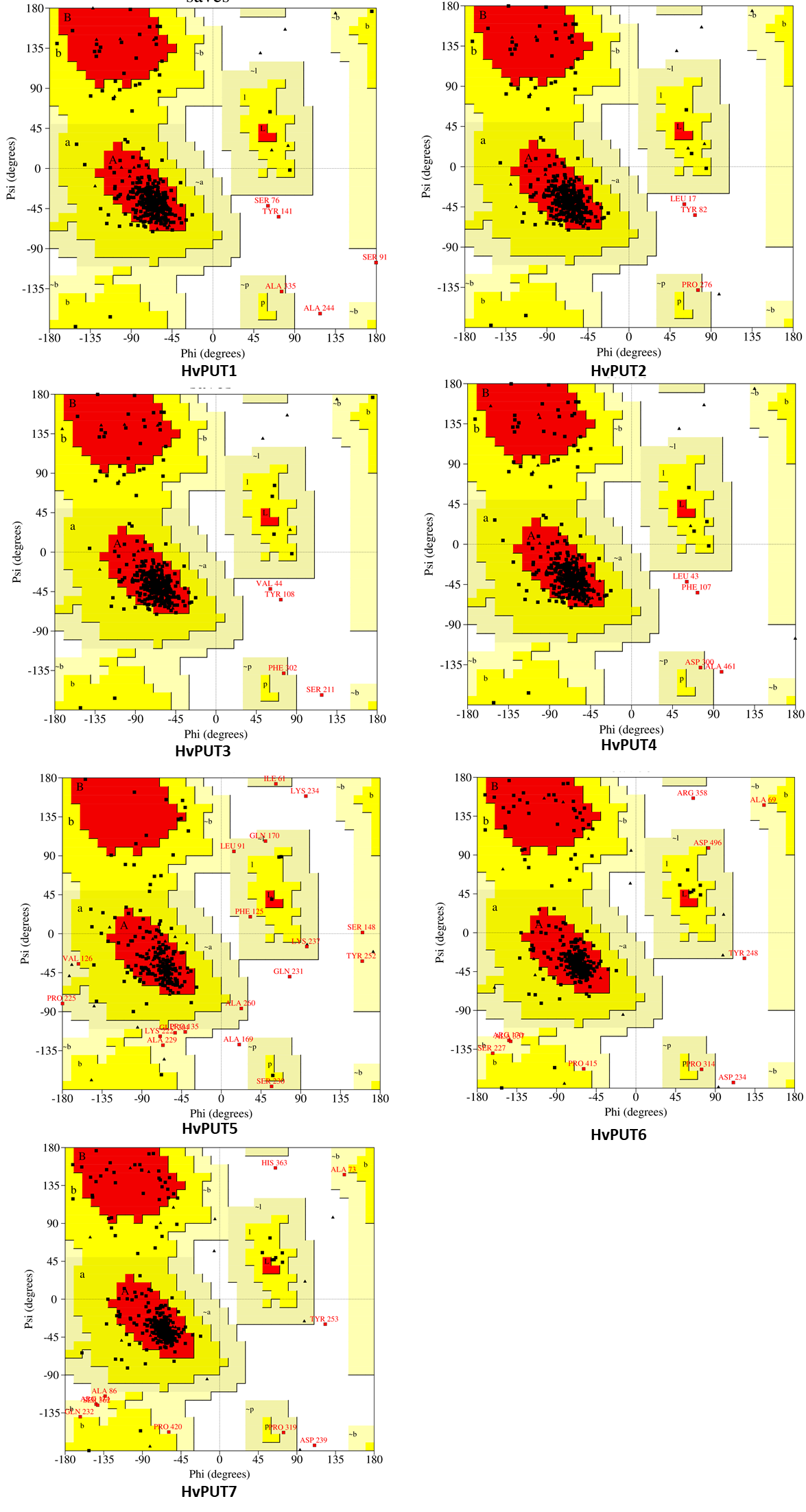


**Supplementary Figure S4.** Ramachandran plot analysis of homology models of HvPUT proteins.


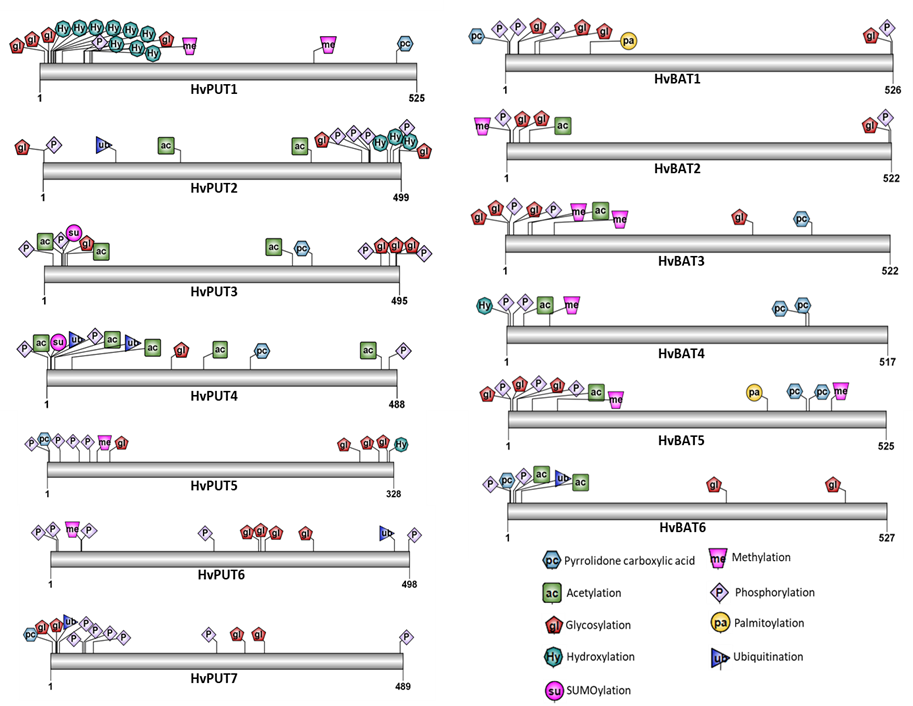


**Supplementary Figure S5. Predicted post-translational modification sites in PA transporter protein sequences of barley.** The bars represent the primary structures of HvBAT and HvPUT proteins, the number of amino acids building the protein is indicated. Nine predicted post-translational modifications namely acetylation, glycosylation, hydroxylation, SUMOylation, methylation, phosphorylation, palmitoylation, ubiquitination, and pyrrolidone carboxylic acid, have been marked in the protein structure.


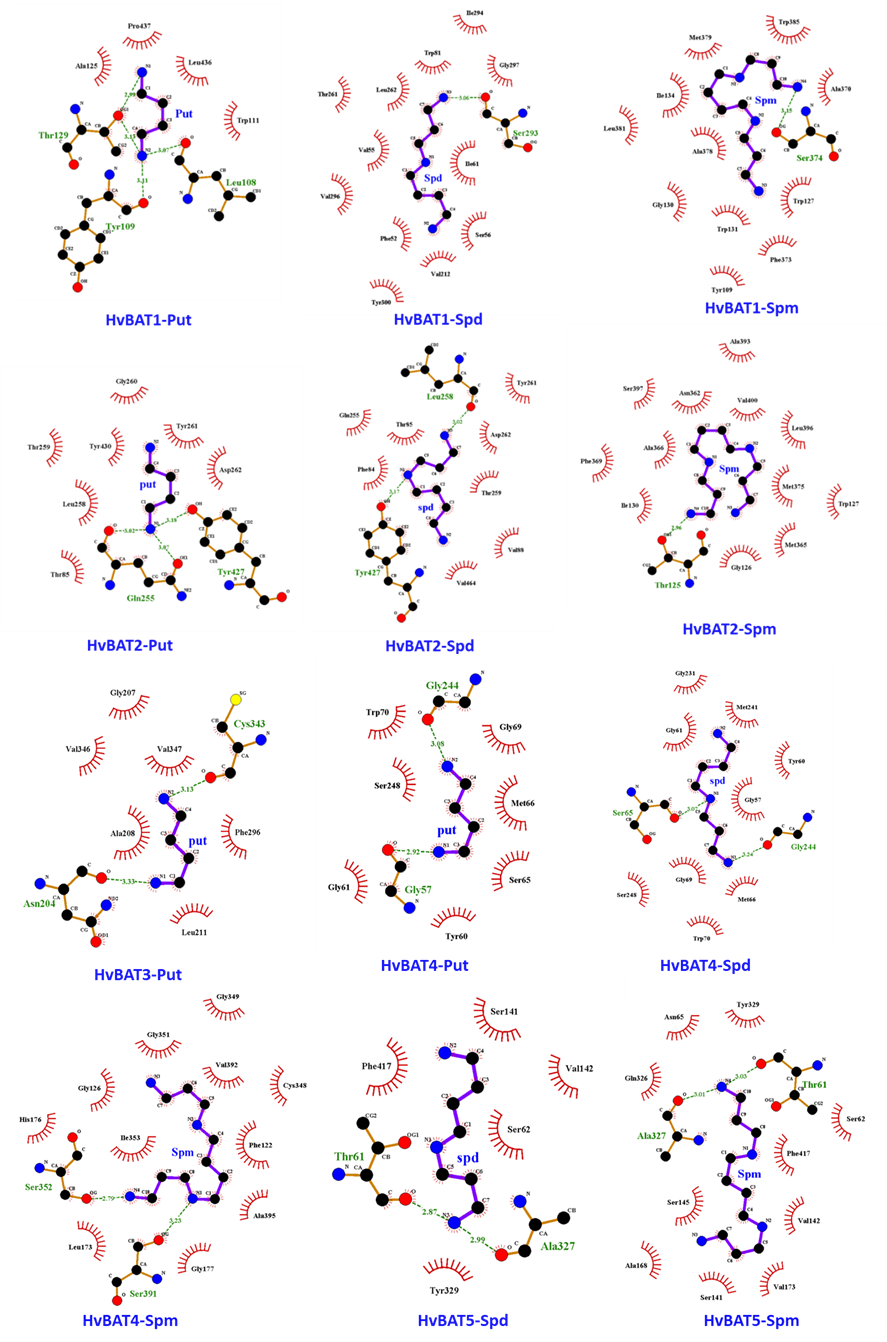


**Supplementary Figure S6.** Interactions of polyamines (PAs) in the active sites of the predicted 3D structures of HvBAT1-5 of barley.


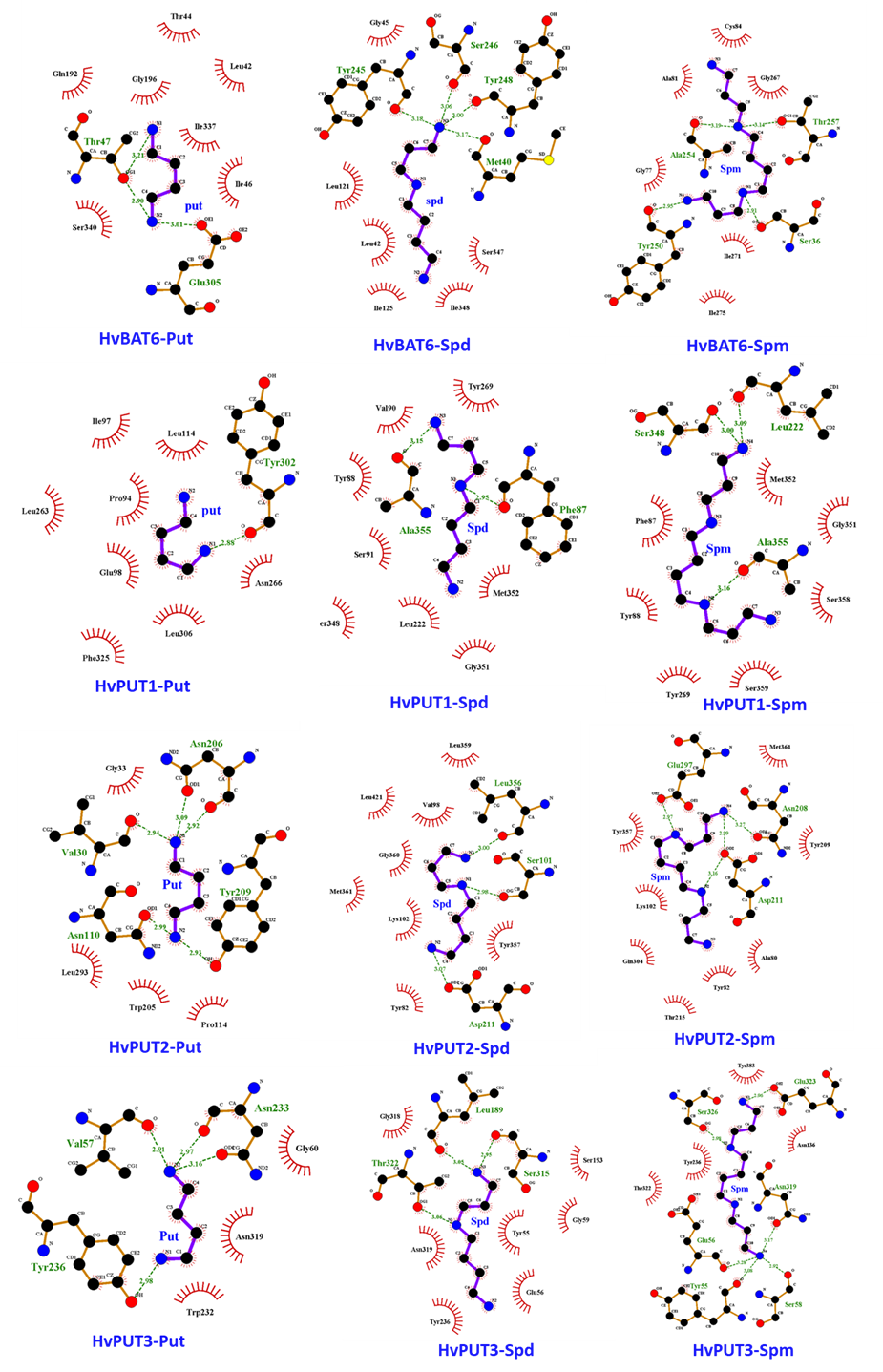


**Supplementary Figure S7.** Interactions of polyamines (PAs) in the active sites of the predicted 3D structures of HvBAT6 and HvPUT1-3 of barley.


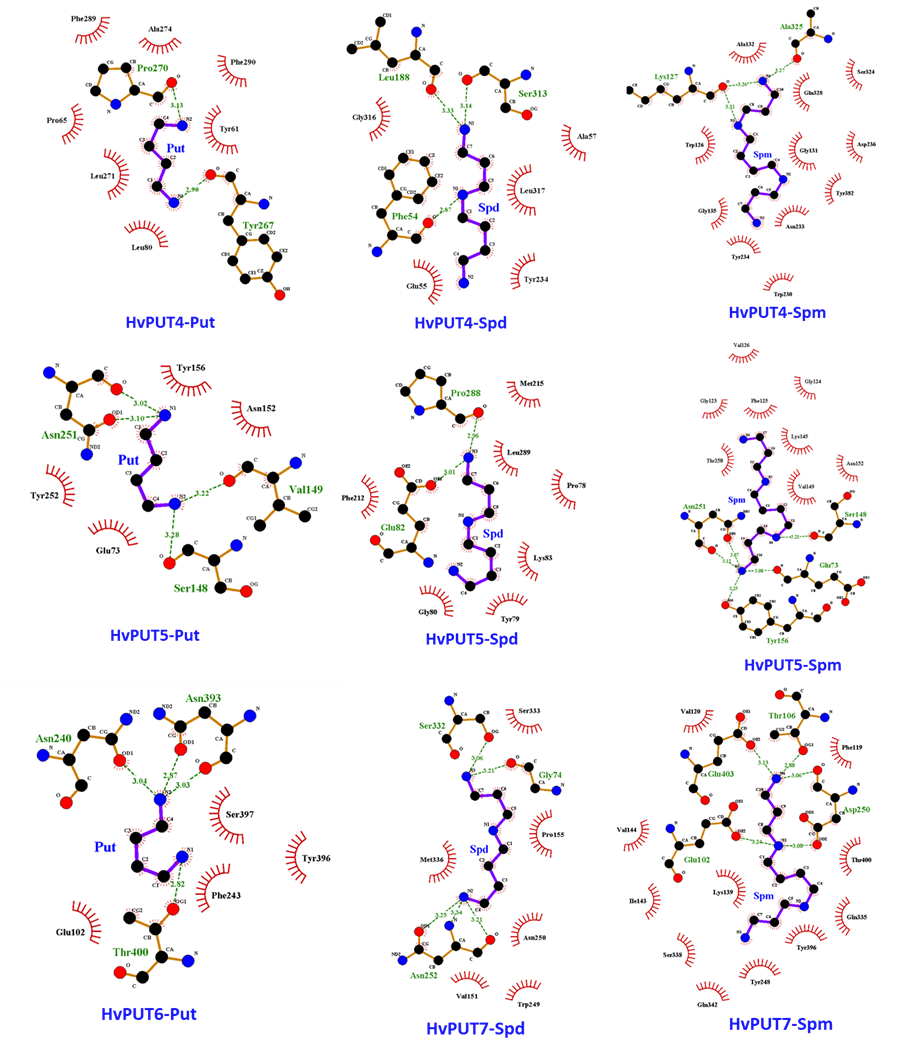


**Supplementary Figure S8.** Interactions of polyamines (PAs) in the active sites of the predicted 3D structures of HvPUT4-7 of barley.
